# Effectiveness of an integrated agriculture, nutrition-specific, and nutrition-sensitive program on child growth in Western Kenya: a cluster-randomized controlled trial

**DOI:** 10.1093/ajcn/nqac098

**Published:** 2022-04-14

**Authors:** Rita Wegmüller, Kelvin Musau, Lucie Vergari, Emily Custer, Hellen Anyango, William E S Donkor, Marion Kiprotich, Kim Siegal, Nicolai Petry, James P Wirth, Sonia Lewycka, Bradley A Woodruff, Fabian Rohner

**Affiliations:** GroundWork, Fläsch, Switzerland; One Acre Fund Headquarters, Kakamega, Kenya; One Acre Fund Headquarters, Kakamega, Kenya; One Acre Fund, Jinja, Uganda; One Acre Fund Headquarters, Kakamega, Kenya; GroundWork, Fläsch, Switzerland; One Acre Fund, Nairobi, Kenya; One Acre Fund, NY, USA; GroundWork, Fläsch, Switzerland; GroundWork, Fläsch, Switzerland; Centre for Tropical Medicine and Global Health, Nuffield Department of Medicine, University of Oxford, UK; Oxford University Clinical Research Unit, Hanoi, Vietnam; GroundWork, Fläsch, Switzerland; GroundWork, Fläsch, Switzerland

**Keywords:** effectiveness, Kenya, growth, children, dietary diversity, morbidity, nutrition, agriculture, WASH, RCT

## Abstract

**Background:**

Stunting rates remain unacceptably high in many regions, including sub-Saharan Africa. Agricultural programs have led to increased yields and household incomes but showed limited success in improving nutritional status.

**Objectives:**

We assessed whether linear growth could be improved through a potentially scalable, integrated program adding nutrition-specific and nutrition-sensitive components to an existing agricultural program.

**Methods:**

In this cluster-randomized controlled trial in rural Western Kenya, we randomized children aged 6–35 months from farming families to an agricultural intervention without (control group) or with a bundle of interventions (intervention group), including distribution of micronutrient powders (MNP), poultry to increase egg consumption, seeds of greens and onions, and soap and chlorine solution, as well as provision of monthly behavior change trainings. The primary outcome was the change in height-for-age *z*-score (HAZ) over 2 years of follow-up. We assessed safety through active morbidity and passive adverse event monitoring. We conducted an intention-to-treat analysis, followed by per-protocol and prespecified subgroup analyses.

**Results:**

From March to April 2018, we enrolled 1927 children from 126 clusters (control, 942 children in 63 clusters; intervention, 985 children in 63 clusters). Data on HAZ were available for 1672 (86.6%) children after 2 years. Adherence was >80% for use of MNP, chlorine, and greens and receipt of soap, and ∼40% for egg and red onion consumption. The intention-to-treat analysis indicated a greater change in HAZ over 2 years in the intervention group (adjusted effect size, 0.11; 95% CI: 0.02–0.19). We found a slightly stronger effect in the per-protocol analysis (adjusted effect size, 0.15; 95% CI: 0.06–0.24). Dietary diversity and consumption of iron-rich foods were improved in the intervention group, and reported instances of fever, lower respiratory tract infections, and diarrheal episodes were lower in the intervention group.

**Conclusions:**

This study found a modest improvement in linear growth, indicating the need for multiple, integrated interventions to achieve benefits. The trial was registered with clinicaltrials.gov as NCT03448484.

## Introduction

Child stunting continues to affect a large proportion of children younger than 5 years (1). Though there has been some progress in reducing the stunting prevalence (2), it has been too slow to achieve the Sustainable Development Goals by 2025 (3). Estimates of the stunting prevalences among children younger than 5 years declined from 33% in 2000 to 22% in 2020 globally, and from 42% to 31% in Africa (4). The multifactorial etiology of stunting (5) may be a reason for this slow progress. A combination of interventions improving the underlying causes, such as poor nutrition and hygiene, have been repeatedly posited to alleviate the problem (2).

Nutrition-specific interventions alone, such as consumption of micronutrient powders (MNP) or lipid-based nutrient supplements, have been shown to reduce anemia and micronutrient deficiencies, but have mixed effects on improving child growth (6, 7). The impact of egg provision on linear growth is mixed; 2 studies providing eggs to young Ecuadorian and Ethiopian children found strong effects on linear growth (8, 9), but a study in Malawi found no impact on growth (10). Practitioners and researchers have traditionally assumed that water, sanitation, and hygiene (WASH) interventions improve child growth (11), but a recent series of well-designed intervention trials have failed to demonstrate improved child growth in the WASH groups, including 1 trial in Western Kenya (12).

In Kenya, 26% of children less than 5 years of age are stunted, and only about a third of households have washing places at their homes (13). Also, nationally, 3 out of 4 households share toilets with other households or have unimproved toilets (open defecation, bucket latrine, etc.). Undernutrition is particularly of public health concern in the Western region of Kenya, where the prevalence of adequate dietary diversity and the proportion of households with washing places are lower and the diarrhea prevalence is slightly higher than the national average in young children (13, 14). Both subpar WASH practices and child and maternal malnutrition have independently been identified as leading risk factors contributing to the overall burden of disease in Kenya (15).

Agriculture is the main source of income and food for most rural families in Sub-Saharan Africa, including those in Kenya. Agricultural programs have led to increased yields, staple production, and household incomes, but have had limited success in improving nutritional status (16). A more recent review highlights the challenge of nutrition-sensitive agriculture alone to achieve an impact on stunting, and recommends that such a program needs to be combined with other strategies, such as WASH and nutrient-rich foods (17).

In the present study, we compared changes in linear growth in a group of young children receiving an existing agricultural intervention to those in a group additionally receiving nutrition-specific and nutrition-sensitive interventions in Kakamega County of Western Kenya.

## Methods

### Study design and participants

This study aimed to assess the effectiveness of a bundle of basic agriculture, nutrition-specific, and nutrition-sensitive interventions (intervention) on child growth compared to basic agriculture interventions (control). We implemented this cluster-randomized controlled trial in the subcounties of Lugari and Likuyani of Kakamega County, in Kenya's western region, from March 2018 to April 2020. The One Acre Fund program (18) was delivering an agricultural bundle to farmers in all 11 wards of the 2 subcounties who were willing to participate. We conducted the study in all 6 wards of Lugari and in 3 neighboring wards in Likuyani. One Acre Fund divided these wards into 126 study clusters. Clusters mainly consisted of 1 village, half a village, or 2 neighboring villages combined, in order to have an average cluster size of approximately 20 potentially eligible participants based on a farmer's household listing. We used these 126 clusters as the units of intervention in the study. Children aged 6–35 months from farming households participating in the One Acre Fund program (providing the agriculture bundle) whose legal guardian provided written informed consent to a One Acre Fund field staff member, were eligible to take part and were enrolled in the study if the family intended to stay within the study area for the following 24 months and the child had no visible, severe disease.

We informed caregivers that they were free to withdraw their children at any time. Children with severe acute malnutrition were referred for treatment but kept in the study under close supervision of the study clinician. We trained and encouraged participating caregivers to report adverse events to the study medical team by phone calls. The medical team arranged for paid transportation and initial consultation at a nearby health facility and made medical follow-up visits to understand the diagnoses of adverse events and determine whether they were related to the study interventions in any way. The medical team closely monitored children with adverse events until resolution. All clinical reports were submitted to the respective ethical and regulatory committees for review and documentation.

The study protocol and subsequent amendments were approved by Swissethics, Zurich, Switzerland (approval number 2017–02007); Amref Health Africa, Nairobi, Kenya (AMREF-ESRC P419/2017); and the Kenya Pharmacy and Poisons Board (PPB/ECCT/19/04/04/2019). Amendments after the study start concerned the change in the composition of the research team and other minor amendments, and were approved by both ethical boards. The study protocol can be accessed at https://osf.io/n8bmf/. We registered the study with clinicaltrials.gov as NCT03448484.

### Randomization and masking

One of the trial epidemiologists randomly allocated clusters to the treatment or control condition using random treatment allocation in Stata (version 16; StataCorp). Allocation was stratified by the 9 wards, and misfits were allocated while prioritizing balance across the 2 groups. There were 4 misfits, 1 each from 4 different strata, and these were randomly assigned, 2 to the intervention group and 2 to the control group, maintaining the overall balance (for details, refer to the **Supplemental Methods**).

Anthropometry data collectors were masked to participants’ group allocation. We tried to keep all other data collectors unaware of the intervention allocation, although the interviewers could have inferred group allocation from certain answers. The main data analyst was not provided with the keys to the group assignments before completion of programming for the analysis of primary outcomes.

### Procedures

We pilot tested the intervention bundle in nonstudy clusters in a different county ahead of the actual study to assess feasibility and acceptability. The results indicated that the intervention products were well accepted and consumed by the participants but that a more detailed intervention product delivery model addressing all stages of product movement was needed and that clusters needed to be geographically separated to prevent spillover of products.

All participating households (both control and intervention groups) had previously registered for the One Acre Fund program and ordered at least 1 of the following products within the agriculture bundle on credit using the standard One Acre Fund repayment approach (19): compost booster; cook stoves; onion, maize, indigenous greens, and bean seeds; maize storage bags; drying tarps; trees; solar lights; fertilizer; actellic dust (insecticide); and reusable sanitary pads. Additionally, we provided agricultural training to all participating households every 2 weeks on average.

Caregivers of intervention children additionally received MNP (MixMe brand; 15 micronutrient formulation (20); DSM) and oral rehydration solution (ORS; 20.6 g; Dawa Lyte, Dawa Ltd) plus zinc (10 mg/tablet; JuniorZinc, Dawa Ltd) for treatment of acute diarrhea. Every month, we distributed 10 MNP sachets, and reminded caregivers to give 1 sachet every 3 days; we resupplied ORS and zinc if caregivers ran out of stock. All households in the intervention group received 600 g of soap every month, 500 ml of chlorine solution every 6 months (Aquaguard, Supersleek Ltd), and 8 laying hens at enrollment. We resupplied intervention households with another 8 hens between December 2018 and June 2019 to compensate for losses. We distributed seeds and fertilizer for red onion and indigenous greens at the beginning of the planting season in March 2018 and March 2019. We encouraged caregivers to feed 1 egg per day and red onions and greens to the participating child. The intervention bundle beyond the basic agriculture package was provided free of charge, and was distributed by One Acre Fund nutrition trainers.

Intervention product distributions were accompanied by monthly nutrition and WASH group trainings, along with product-specific instructions for use (for details, refer to the Supplemental Methods). We based these trainings on materials such as pictorial brochures, storybooks, and toys, leveraging social behavior change communication techniques to engage caregivers and foster discussion. Modules included a wide range of topics, including dietary diversity, nutrition and care for sick children, water treatment and storage, and use of MNPs.

We trained study personnel on study-specific procedures before the baseline and prior to subsequent assessment rounds. For anthropometrists, repeat standardization exercises were included. Within a week of obtaining written informed consent, we conducted the round 1 (baseline) assessment, followed by the round 2 assessment 1 year after round 1, and the round 3 assessment 2 years after round 1. During these assessment rounds, we took anthropometric measurements and interviewers asked caregivers about their child's dietary diversity, infant and young child feeding practices, WASH practices, recent child morbidity, and adherence to the intervention bundle. Additionally, in round 1, we collected basic demographic information, including child birthdate and sex, household food security, household wealth indicators, and caregiver's age and education. In addition to the main assessment rounds, we conducted quarterly adherence and morbidity monitoring. Questionnaire data were collected using tablets with preinstalled CommCare data collection software (Dimagi).

We conducted anthropometry measurements at a central place within the cluster, and 2 anthropometry teams working independently measured participating children; results were recorded on paper forms and then directly entered into the tablet in order to compare the measurements of the 2 teams. If the 2 length or height measurements differed by more than 0.7 cm or the weight measurements differed by more than 0.1 kg, both teams repeated the anthropometric measurements for this child until measurements were within the acceptable range. Both weight and length or height were measured using standard procedures (21), using Seca scales (model 857) and UNICEF height boards (item number S0114540). We calibrated the scales daily with standard weights. For children not able to stand by themselves, we first weighed the caregiver alone, then together with the child using the scale's tare function. For children under 2 years of age, we measured recumbent length. Older children were measured standing.

### Outcomes

We hypothesized that linear growth in children 6–35 months of age at enrollment would improve after the provision of the intervention bundle over a 2-year period when compared to the control group. The primary outcome was the change in height-for-age *z*-score (HAZ) over the 2 years of follow-up. The primary analysis used an intention-to-treat approach at the individual participant level.

Secondary objectives were set to determine, at the individual participant level, the positive and negative effects of the intervention, and included the following set of indicators to be compared between the intervention and control groups at 1 year and 2 years of follow-up: the changes in weight-for-age *z*-score (WAZ) and weight-for-height *z*-score (WHZ); the proportions of children stunted, underweight, wasted, overweight, with continued breastfeeding, with minimum dietary diversity, and with recent diarrhea, fever, and lower respiratory tract infection (LRTI).

### Sample size and statistical analysis

The primary outcome was the change in HAZ between enrollment and the 2-year follow-up. We calculated a minimum sample size of 933 children to be recruited into each of the 2 study groups based on a minimum detectable effect size of 0.17 in the average difference in HAZ between intervention and control groups; this effect size has been shown in other studies (22). This sample size calculation also assumed a power of 0.8, a 2-sided alpha of 0.05, and a design effect of 1.3 due to cluster randomization. We assumed that caregivers of 95% of eligible children would consent to participation and that 80% of enrolled children would complete the full 2 years of follow-up. We slightly inflated the sample size to a round target number of 1000 children per group. This sample would be recruited in 126 clusters, stratified by wards.

Two epidemiologists independently conducted the intention-to-treat analysis of the primary outcome by using IBM SPSS Statistics (version 27) and Stata (version 16).

All hypothesis testing, including the calculation of all measures of precision and statistical significance, was carried out accounting for the stratified and clustered design. The primary outcome was normally distributed, and residual plots raised no concerns about normal assumptions. Quantile-Quantile plots suggested long tails with sparse data, which were addressed through our 2 sensitivity analyses (see below). For the primary outcome of HAZ and the secondary outcomes of WAZ and WHZ, we tested the statistical significance of the mean change using mixed-effects, generalized linear models with treatment group and strata as fixed effects and cluster as a random effect to compare the average changes in the intervention and the control groups during the follow-up period. We conducted separate analyses for the first year of follow-up, the second year of follow-up (both secondary), and the entire 2-year follow-up period (primary). Adjusted models additionally included baseline values and used child age and sex as fixed effects; for the change in WAZ, the wealth index was also included. Factors were selected a priori based on known associations with growth, and factors included in the final models were those found to be associated with the outcome in bivariate analyses. Age and sex were prespecified as potential effect modifiers, and subgroup analyses were conducted. We tested heterogeneity between subgroups using likelihood ratio tests. We compared changes in prevalence, such as for stunting and wasting, between groups at each time point using a mixed-effects Poisson regression analysis with treatment group and strata as fixed effects and cluster as a random effect to generate relative risks. Child age and sex were included as additional fixed effects in an adjusted analysis. A mixed-effects Poisson regression was also used to compare incidences of adverse events, with treatment group as a fixed effect and cluster as a random effect.

To create an overall adherence index to the different interventions for each child, we first calculated adherence for each intervention separately and then ran a principal component analysis with key adherence indicators included in the model (for details, refer to the Supplemental Methods) to produce a combined adherence index. For the per-protocol analysis, children in the lowest tercile of this adherence index were excluded from analysis.

## Results

**FIGURE 1:**
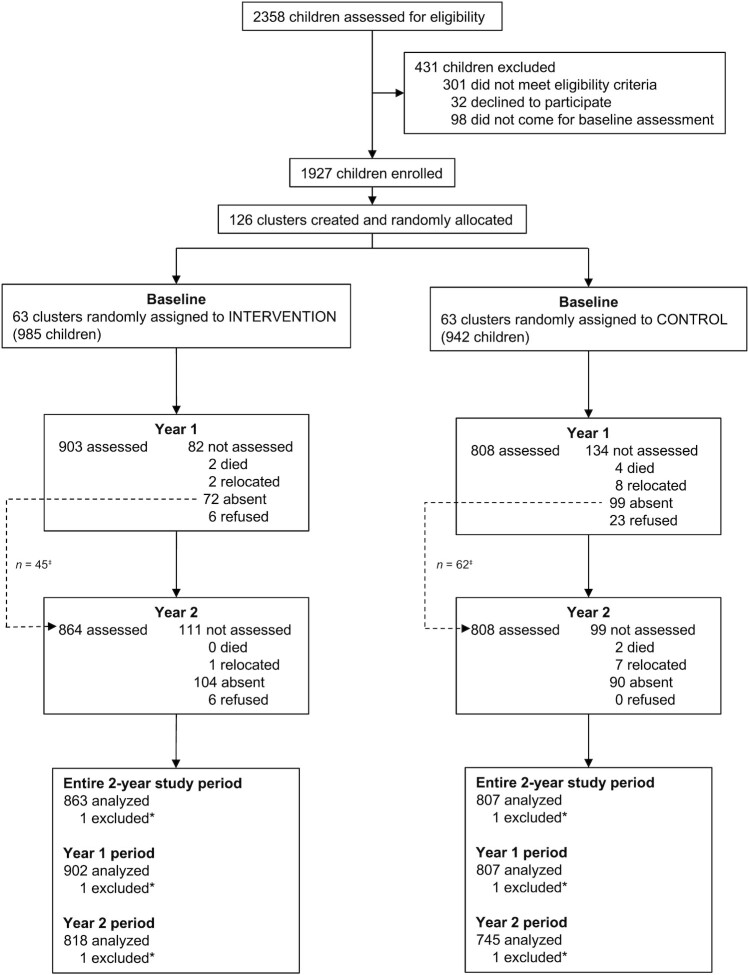
Trial profile and analysis populations for primary outcome. ^‡^Participants absent at the year-1 follow-up were eligible to complete the year-2 follow-up and were included in the entire 2-year study period analysis, but not in the year-1 period and year-2 period analyses due to missing data at the year-1 follow-up. *Excluded due to an implausible date of birth after investigation.

Between March and April 2018, we enrolled 1927 children from 126 clusters into the study (**Figure 1**). Of the 985 children recruited in intervention clusters, 92% were available for assessment at the year-1 follow-up and 88% at the year-2 follow-up. Of the 942 children recruited in control clusters, we assessed 86% at both the year-1 and year-2 follow-ups. Full details on participant exclusion and losses to follow-up are shown in Figure 1. The proportions of missing data for the primary outcome did not differ between the intervention and control groups (12% and 14%, respectively).

Baseline characteristics for all participants and for those with complete outcome data were similar across the 2 groups (**Table 1**); the variable with the greatest difference was the proportion of children living in households with access to safe drinking water, which was higher in the intervention areas. In slightly more than one-half of study participants, the primary caregiver was the biological mother. The mean participant age at recruitment was 21.8 months, and half of recruited children were female. Few children lived in households with electricity, but most drank safe water. Less than one-third had improved sanitation. Moderate and severe food insecurity were found in almost 90% of study children's households.

**TABLE 1:** Child, household, and caregiver baseline characteristics of study children by intervention group in the intention-to-treat population^1^

	All children		Children with primary outcome data	
Characteristics	Intervention (*n* = 984)	Control (*n* = 941)	Intervention (*n* = 862)	Control (*n* = 807)
Child				
Mean age (SD), months	21.5 (8.5)	22.0 (8.5)	21.8 (8.5)	22.1 (8.5)
Male sex	478/984 (49%)	474/941 (50%)	423/862 (49%)	402/807 (50%)
Caregiver is biologic mother	539/984 (55%)	514/941 (55%)	481/862 (56%)	448/807 (56%)
If not mother, relationship to child				
Grandmother	413/445 (93%)	388/427 (91%)	356/381 (93%)	327/359 (91%)
Aunt	20/445 (4%)	23/427 (5%)	16/381 (4%)	19/359 (5%)
Other relative	12/445 (3%)	16/427 (4%)	9/381 (2%)	13/359 (4%)
Mean height-for-age *z*-score (SD)	−1.06 (1.36)	−1.13 (1.28)	−1.04 (1.35)	−1.12 (1.27)
Mean weight-for-age *z*-score (SD)	−0.56 (1.14)	−0.59 (1.07)	−0.55 (1.13)	−0.58 (1.05)
Mean weight-for-height *z*-score (SD)	−0.02 (0.99)	−0.02 (0.97)	−0.02 (0.98)	−0.01 (0.97)
Micronutrient powders ever used	11/983 (1.1%)	8/938 (0.9%)	10/861 (1.2%)	5/804 (0.6%)
Early initiation of breastfeeding (6–23 months of age)	403/571 (71%)	382/514 (74%)	333/485 (69%)	326/435 (75%)
Iron syrup in previous 6 months (6–23 months of age)	49/564 (9%)	31/502 (6%)	39/479 (8%)	21/424 (5%)
Vitamin A in previous 6 months (6–23 months of age)	448/557 (80%)	419/502 (83%)	379/473 (80%)	359/426 (84%)
Anthelmintics in previous 6 months (6–23 months of age)	190/559 (34%)	174/502 (35%)	160/476 (34%)	152/426 (36%)
Household				
Mean number of household members (SD)	6.7 (2.2)	6.9 (2.6)	6.7 (2.2)	7.0 (2.6)
Mean number of persons per sleeping room (SD)	3.5 (1.7)	3.4 (1.6)	3.5 (1.7)	3.5 (1.6)
Household head				
Caregiver	155/984 (16%)	138/941 (15%)	138/862 (16%)	112/807 (14%)
Partner	757/984 (77%)	711/941 (76%)	669/862 (78%)	621/807 (77%)
Other	72/984 (7%)	92/941 (10%)	55/862 (6%)	74/807 (9%)
Has electricity	101/984 (10%)	95/940 (10%)	81/862 (9%)	83/807 (10%)
Mean wealth index (SD)	0.04 (1.03)	−0.04 (0.96)	0.03 (1.03)	−0.07 (0.96)
Safe water source^2^	776/984 (79%)	686/940 (73%)	678/862 (79%)	585/807 (72%)
Mean time to fetch water (SD), minutes	17.6 (15.3)	17.0 (15.5)	17.9 (15.6)	17.0 (15.9)
Drink safe water^2^	917/984 (93%)	829/940 (88%)	802/862 (93%)	707/807 (88%)
Improved sanitation^2^	294/982 (30%)	245/936 (26%)	253/860 (29%)	205/803 (26%)
Food insecurity^3^				
None	54/984 (5%)	67/941 (7%)	47/862 (5%)	59/807 (7%)
Mild	53/984 (5%)	58/941 (6%)	41/862 (5%)	48/807 (6%)
Moderate	380/984 (39%)	358/941 (38%)	341/862 (40%)	309/807 (38%)
Severe	497/984 (51%)	458/941 (49%)	433/862 (50%)	391/807 (48%)
Mother or caregiver				
Mean age (SD), years	39.8 (13.2)	38.4 (12.9)	39.9 (13.2)	38.3 (12.8)
Marital status				
Married	828/983 (84%)	783/940 (83%)	724/861 (84%)	681/807 (84%)
Widowed	83/983 (8%)	83/940 (9%)	77/861 (9%)	67/807 (8%)
Single	45/983 (5%)	54/940 (6%)	39/861 (5%)	44/807 (5%)
Separated/divorced	27/983 (3%)	20/940 (2%)	21/861 (2%)	15/807 (2%)
Educational status				
None	44/983 (4%)	35/940 (4%)	39/861 (5%)	32/806 (4%)
Primary	592/983 (60%)	563/940 (60%)	529/861 (61%)	478/806 (59%)
Postprimary	14/983 (1%)	5/940 (1%)	11/861 (1%)	5/806 (1%)
Secondary	290/983 (30%)	308/940 (33%)	247/861 (29%)	264/806 (33%)
College/University	43/983 (4%)	28/940 (3%)	35/861 (4%)	27/806 (3%)
Prior pregnancy (where the mother is the caregiver)	455/538 (85%)	427/514 (83%)	412/481 (86%)	374/448 (83%)
Mean number of prior live births (where the mother is the caregiver) (SD)	3.2 (1.9)	3.1 (1.9)	3.3 (2.0)	3.2 (1.9)

1 Data are *n* (%) for categorical variables or mean (SD) for continuous variables.

2 Defined by the WHO and UNICEF Joint Monitoring Program's definitions.

3 Assessed by the Food and Nutrition Technical Assistance Household Food Insecurity Access Scale.

Individual adherence indicators are shown in **Table 2**. Intervention product consumption was generally high, except for children's egg and household's red onion consumption, which were less than 50% of the target. Despite the lower adherence to these 2 products, children in the intervention group consumed more eggs on average than the control group (2.8 vs. 0.6 eggs per week, respectively), and 46% of intervention households consumed red onions from their own harvest during both study years, compared to only 1% in the control group. Household consumption of self-grown greens during both study years and treatment of diarrheal episodes with zinc and/or ORS were below 50% in the control group, but above 80% in the intervention group. As expected, few control children consumed MNP. Adherence to the monthly behavior change trainings was 95% in the intervention group.

**TABLE 2:** Adherence indicators and use of intervention products by study group during both years, year 1 and year 2^1^

Indicator	Targets for intervention group	Intervention group	Control group
Proportion of caregivers interviewed (% monitoring coverage)			
Both years	100%	907/984 (92%)	818/941 (87%)
Year 1	100%	916/984 (93%)	820/941 (87%)
Year 2	100%	899/984 (91%)	816/941 (87%)
Mean (SD) number of eggs consumed in past 7 days^2^			
Both years	7	2.8 (1.4)	0.6 (0.6)
Year 1	7	3.3 (1.6)	0.5 (0.7)
Year 2	7	2.2 (1.6)	0.6 (0.7)
Mean (SD) number of MNP sachets consumed^3^			
Both years	240	197.1 (8.8)	0.3 (2.9)
Year 1	120	112.6 (6.1)	0.2 (1.9)
Year 2	120	84.5 (5.7)	0.1 (1.5)
Mean (SD) weight of project-provided chlorine solution used per 3 months^4^			
Both years	275 g	292.9 (99.3)	NA^5^
Year 1	275 g	267.9 (124.5)	NA
Year 2	275 g	322.7 (126.7)	NA
Mean (SD) number of quarters in which receipt of soap was reported			
Both years	8	7.3 (1.2)	NA
Year 1	4	3.7 (0.6)	NA
Year 2	4	3.7 (0.7)	NA
Proportion consuming onions from own harvest^6^			
Consumed in both years (%)	100%	449/984 (46%)	9/931 (1%)
Not consumed at all (%)		159/984 (16%)	835/931 (90%)
Consumed in first or second year (%)		376/984 (38%)	87/931 (9%)
Proportion consuming greens from own harvest^6^			
Consumed in both years (%)	100%	822/984 (84%)	420/931 (45%)
Not consumed at all (%)		20/984 (2%)	147/931 (16%)
Consumed in first or second year (%)		142/984 (14%)	364/931 (39%)
Proportion of reported diarrhea episodes for which child received zinc supplement^7^			
Both years	100%	453/539 (84%)	146/562 (26%)
Year 1	100%	301/363 (83%)	103/390 (26%)
Year 2	100%	152/176 (86%)	43/172 (25%)
Proportion of reported diarrhea episodes for which child received ORS^7^			
Both years	100%	465/539 (86%)	228/562 (41%)
Year 1	100%	308/363 (85%)	163/390 (42%)
Year 2	100%	157/176 (89%)	65/172 (38%)
Mean (SD) number of nutrition and WASH trainings attended by either caregiver or representative^8^			
Both years	26	24.6 (2.0)	NA
Year 1	13	12.0 (1.5)	NA
Year 2	13	12.6 (1.0)	NA

1 Data are *n* (%) for categorical variables or mean (SD) for continuous variables. Year-1 and year-2 data are composed of 4 quarterly monitoring assessments, and both-year data combine all assessments (*n* = 8). Abbreviations: MNP, micronutrient powders; NA, not applicable; ORS, oral rehydration solution; WASH, water, sanitation, and hygiene.

2 Caregiver-reported data for the number of eggs consumed in past 7 days were collected every quarter.

3 Assessed by counting of empty and full sachets every month.

4 One bottle of 550 g was distributed every 6 months. Due to earlier-than-scheduled distribution in part of the households, we only used the monitoring results of the first quarter after distribution (3 months after distribution); thus, consumption in some households was more than half a bottle (more than 275 g).

5 Chlorine and soap use were not assessed in a comparable way in the control group, and control group caregivers did not receive any nutrition and WASH trainings.

6 Caregiver-reported data for household consumption of the vegetables grown from the seeds received by the study in the intervention group and grown from own seeds in the control group.

7 This was not included in the adherence index because the denominator (number of diarrhea cases) is considered a dependent variable and, as such, yielded a changing and reduced number over time.

8 This was not included in the adherence index, as it was considered an intermediary measure that should mainly result in improved adherence and some of the secondary outcomes. Trainings were attended by either the caregiver or representative; the majority of trainings (82%) were attended by the caregiver.

The adjusted intention-to-treat analyses demonstrate larger increases in HAZ in intervention children than in control children during the entire 2-year follow-up (effect, 0.11; 95% CI: 0.02–0.19) and during the first year of follow-up alone (effect, 0.07; 95% CI: 0.002–0.13; **Table 3**). Differences in average changes in HAZ between intervention and control children during the second year of follow-up were much smaller and not statistically significant. The differences in average changes in WAZ were minimal between intervention and control children during the entire 2-year follow-up (effect, 0.03; 95% CI: −0.03 to 0.08). During the second year of follow-up, the WHZ remained the same in intervention children, but increased in control children. The results of the per-protocol analyses were qualitatively similar, with a slightly stronger effect for changes in HAZ over the entire 2-year follow-up period (effect, 0.15; 95% CI: 0.06–0.24) than in intention-to-treat analyses (**Supplemental Table 1**). We conducted 2 sensitivity analyses for changes in HAZ, WAZ, and WHZ. The first excluded all changes in *z*-scores ≤−4 and ≥4. The second recoded values <−4 to −4 and values >4 to 4. We also compared *z*-scores at the endpoint for comparability with other studies. All 3 yielded comparable results to those presented in Table 3 (data for HAZ are shown in **Supplemental Table 2**). Unadjusted mean *z*-scores at baseline and the year-1 and year-2 follow-ups are presented in **Supplemental Table 3**.

**TABLE 3:** Average changes in z-scores for intervention and control children during both years of follow-up, during the year-1 follow-up, and during the year-2 follow-up, using intention-to-treat analyses^1^

	*n* ^2^	Unadjusted mean change (95% CI)	*n*	Unadjusted mean change (95% CI)		Adjusted mean change (95% CI)		
	Intervention		Control		Effect (95% CI)	Intervention	Control	Effect (95% CI)
Change in HAZ								
Both years	862	0.25 (0.19–0.31)	807	0.17 (0.11–0.24)	0.07 (−0.02 to 0.17)	0.26 (0.21–0.32)	0.16 (0.10–0.22)	0.11 (0.02–0.19)^3^
Year 1	901	0.12 (0.07–0.16)	807	0.06 (0.01–0.11)	0.06 (−0.01 to 0.13)	0.12 (0.08–0.17)	0.05 (0.01–0.10)	0.07 (0.002–0.13)^3^
Year 2	816	0.12 (0.07–0.18)	745	0.11 (0.05–0.16)	0.02 (−0.05 to 0.09)	0.13 (0.08–0.18)	0.10 (0.05–0.16)	0.02 (−0.05 to 0.09)
Change in WAZ								
Both years	863	−0.02 (−0.06 to 0.03)	807	−0.03 (−0.08 to 0.02)	0.01 (−0.05 to 0.08)	−0.01 (−0.05 to 0.03)	−0.04 (−0.08 to 0.005)	0.03 (−0.03 to 0.08)
Year 1	902	−0.07 (−0.11 to −0.03)	807	−0.10 (−0.15 to −0.06)	0.04 (−0.02 to 0.10)	−0.06 (−0.10 to −0.03)	−0.11 (−0.14 to −0.07)	0.04 (−0.01 to 0.10)
Year 2	818	0.05 (0.01–0.08)	745	0.07 (0.04–0.11)	−0.03 (−0.08 to 0.02)	0.05 (0.01–0.08)	0.08 (0.04–0.11)	−0.03 (−0.08 to 0.02)
Change in WHZ								
Both years	860	−0.15 (−0.19 to −0.10)	806	−0.09 (−0.14 to −0.05)	−0.05 (−0.12 to 0.01)	−0.15 (−0.19 to −0.11)	−0.09 (−0.13 to −0.05)	−0.06 (−0.11 to −0.001)^3^
Year 1	902	−0.16 (−0.20 to −0.11)	807	−0.16 (−0.20 to −0.11)	−0.001 (−0.07 to 0.07)	−0.16 (−0.20 to −0.11)	−0.15 (−0.20 to −0.11)	−0.001 (−0.06 to 0.06)
Year 2	815	0.01 (−0.03 to 0.04)	744	0.07 (0.03–0.11)	−0.07 (−0.12 to −0.01)^3^	0.01 (−0.03 to 0.04)	0.07 (0.03–0.11)	−0.07 (−0.12 to −0.01)^3^

1 Mixed-effects generalized linear models with treatment groups and strata as fixed effects and the cluster as the random effect were used to compare the unadjusted average changes in the intervention and the control groups during the follow-up period. In the adjusted analyses, we included the baseline *z*-score as a fixed factor in all models. In addition, of the prespecified baseline variables considered for inclusion in adjusted analyses (child age, child sex, whether the caregiver was the biological mother, caregiver's age, caregiver's education, caregiver's marital status, wealth index, household food insecurity index, number of household members, mean time to get water, drinking of safe water, improved sanitation), only child age and child sex were included as fixed effects in multivariable analyses for HAZ and WHZ due to their significant predictions of changes in HAZ and WHZ in bivariate analyses. For WAZ, the wealth index was additionally included as a fixed effect, as it was a significant predictor in bivariate analyses. Abbreviations: HAZ, height-for-age *z*-score; WAZ, weight-for-age *z*-score; WHZ, weight-for-height *z*-score.

2 Slight differences in numbers between different z-scores are due to some flagged values that were excluded for analyses.

3 Significant effect at the 0.05 level.

Our prespecified subgroup analyses did not indicate any effect modification, but these tests were underpowered to detect the modest differences that might be expected if there were true subgroup effects (all *P* values for interactions > 0.1). Stratum-specific, adjusted intention-to-treat analyses showed significantly greater increases in HAZ in younger intervention children than in younger control children during the entire 2-year follow-up period and during the first year of follow-up alone (**Table 4**). Additionally, younger intervention children showed a lower decline in WAZ than younger control children during the first year of follow-up. In contrast, younger control children showed a smaller decline in WHZ than intervention children during the entire 2-year follow-up period, and a larger increase during the second year of follow-up. Older children showed little differences in changes in HAZ, WAZ, and WHZ between the intervention and control groups. A subgroup analysis by sex showed mixed results: girls in the intervention group had a significantly greater increase in HAZ than girls in the control group during the entire 2-year follow-up period. In the first year of follow-up, boys in the intervention group showed a greater increase in HAZ compared to boys in the control group. The WHZ in girls receiving the intervention declined in the second year, while it increased in girls in the control group.

**TABLE 4:** Average changes in *z*-scores for intervention and control children by age and sex subgroups during both years of follow-up, during the year-1 follow-up, and during the year-2 follow-up, using adjusted intention-to-treat analyses^1^

	Both years					Year 1					Year 2				
	*n* ^2^	Adjusted mean change (95% CI)	*n*	Adjusted mean change (95% CI)	Effect (95% CI)	*n*	Adjusted mean change (95% CI)	*n*	Adjusted mean change (95% CI)	Effect (95% CI)	*n*	Adjusted mean change (95% CI)	*n*	Adjusted mean change (95% CI)	Effect (95% CI)
Outcome	Intervention		Control			Intervention		Control			Intervention		Control		
Intention-to-treat by age^3^															
Change in HAZ	—	—	—	—	*P* value, 0.138^4^	—	—	—	—	*P* value, 0.202	—	—	—	—	*P* value, 0.995
6–23 months^5^	487	0.19	446	0.04	0.15	515	0.05	443	–0.05	0.10	456	0.12	406	0.10	0.02
	—	(0.11–0.26)	—	(−0.04 to 0.11)	(0.05–0.25)^6^	—	(−0.01 to 0.11)	—	(−0.12 to 0.01)	(0.01–0.19)^6^	—	(0.06–0.18)	—	(0.03–0.16)	(−0.07 to 0.11)
24–35 months^7^	375	0.36	361	0.31	0.04	386	0.21	364	0.19	0.02	360	0.14	339	0.11	0.02
	—	(0.28–0.44)	—	(0.23–0.40)	(−0.07 to 0.16)	—	(0.14–0.28)	—	(0.12–0.26)	(−0.08 to 0.12)	—	(0.07–0.20)	—	(0.04–0.18)	(−0.07 to 0.12)
Change in WAZ	—	—	—	—	*P* value, 0.959	—	—	—	—	*P* value, 0.197	—	—	—	—	*P* value, 0.146
6–23 months	488	−0.09	446	−0.11	0.02	516	−0.12	443	−0.19	0.07	458	0.03	406	0.09	−0.06
	—	(−0.14 to −0.04)	—	(−0.16 to −0.06)	(−0.05 to 0.10)	—	(−0.17 to −0.07)	—	(−0.24 to −0.14)	(0.001–0.14)^6^	—	(−0.02 to 0.07)	—	(0.04–0.13)	(−0.12 to 0.004)
24–35 months	375	0.08	361	0.06	0.03	386	0.01	364	0.001	0.01	360	0.07	339	0.06	0.01
	—	(0.02–0.14)	—	(−0.001 to 0.12)	(−0.06 to 0.11)	—	−0.05 to 0.06)	—	(−0.05 to 0.06)	(−0.07 to 0.08)	—	(0.02–0.12)	—	(0.01–0.11)	(−0.06 to 0.08)
Change in WHZ	—	—	—	—	*P* value, 0.181	—	—	—	—	*P* value, 0.712	—	—	—	—	*P* value, 0.108
6–23 months	487	−0.18	447	−0.09	−0.09	516	−0.19	443	−0.20	0.01	457	0.01	407	0.12	−0.11
	—	(−0.23 to −0.13)	—	(−0.15 to −0.04)	(−0.17 to −0.01)^6^	—	(−0.24 to −0.14)	—	(−0.26 to −0.14)	(−0.07 to 0.09)	—	(−0.04 to 0.06)	—	(0.07–0.17)	(−0.18 to −0.03)^6^
24–35 months	373	−0.10	359	−0.09	−0.01	386	−0.11	364	−0.10	−0.01	358	−0.004	337	0.01	−0.02
	—	(−0.16 to −0.04)	—	(−0.15 to −0.03)	(−0.10 to 0.07)	—	(−0.17 to −0.05)	—	(−0.16 to −0.04)	(−0.10 to 0.07)	—	(−0.06 to 0.05)	—	(−0.04 to 0.07)	(−0.10 to 0.06)
Intention-to-treat by sex^8^															
Change in HAZ	—	—	—	—	*P* value, 0.437	—	—	—	—	*P* value, 0.343	—	—	—	—	*P* value, 0.114
Female	439	0.23	405	0.10	0.13	456	0.08	411	0.04	0.04	411	0.12	377	0.05	0.07
	—	(0.16–0.31)	—	(0.02–0.18)	(0.03–0.24)^6^	—	(0.02–0.14)	—	(−0.02 to 0.11)	(−0.05 to 0.13)	—	(0.06–0.19)	—	(−0.01 to 0.12)	(−0.02 to 0.16)
Male	423	0.29	402	0.22	0.08	445	0.16	396	0.07	0.10	405	0.13	368	0.16	−0.03
	—	(0.22–0.37)	—	(0.14–0.29)	(−0.03 to 0.19)	—	(0.10–0.23)	—	(−0.0004 to 0.13)	(0.01–0.19)^6^	—	(0.07–0.19)	—	(0.09–0.22)	(−0.12 to 0.07)
Change in WAZ	—	—	—	—	*P* value, 0.827	—	—	—	—	*P* value, 0.989	—	—	—	—	*P* value, 0.956
Female	439	−0.08	405	−0.11	0.03	457	−0.10	411	−0.14	0.04	412	0.01	377	0.04	−0.03
	—	(−0.13 to −0.03)	—	(−0.17 to −0.05)	(−0.05 to 0.11)	—	(−0.15 to −0.05)	—	(−0.19 to −0.09)	(−0.03 to 0.11)	—	(−0.04 to 0.05)	—	(−0.01 to 0.08)	(−0.10 to 0.04)
Male	424	0.06	402	0.04	0.02	445	−0.03	396	−0.07	0.04	406	0.09	368	0.12	−0.03
	—	(0.003–0.11)	—	(−0.02 to 0.10)	(−0.06 to 0.10)	—	(−0.08 to 0.02)	—	(−0.12 to −0.01)	(−0.03 to 0.11)	—	(0.04–0.13)	—	(0.07–0.16)	(−0.09 to 0.04)
Change in WHZ	—	—	—	—	*P* value, 0.715	—	—	—	—	*P* value, 0.438	—	—	—	—	*P* value, 0.158
Female	438	−0.22	405	−0.15	−0.07	457	−0.18	411	−0.20	0.02	411	−0.05	377	0.05	−0.11
	—	(−0.28 to −0.17)	—	(−0.21 to −0.10)	(−0.15 to 0.01)	—	(−0.23 to −0.12)	—	(−0.25 to −0.14)	(−0.06 to 0.10)	—	(−0.10 to 0.001)	—	(−0.001 to 0.11)	(−0.18 to −0.03)^6^
Male	422	−0.07	401	−0.03	−0.05	445	−0.13	396	−0.11	−0.02	404	0.06	367	0.09	−0.03
	—	(−0.13 to −0.02)	—	(−0.08 to 0.03)	(−0.13 to 0.04)	—	(−0.19 to −0.08)	—	(−0.17 to −0.05)	(−0.10 to 0.06)	—	(0.01–0.12)	—	(0.04–0.15)	(−0.10 to 0.05)

1 To obtain the subgroup-specific estimates for age and sex, we created new indicator variables using reparameterization. Abbreviations: HAZ, height-for-age *z*-score; WAZ, weight-for-age *z*-score; WHZ, weight-for-height *z*-score.

2 Slight differences in numbers between different *z*-scores are due to some flagged values that were excluded for analysis.

3 In the adjusted mixed-effects generalized linear regression models, treatment group, strata, baseline *z*-score, and child sex were included as fixed effects and cluster as a random effect in all models, and wealth index was additionally included in the WAZ model.

4 *P* values from likelihood ratio tests between subgroups.

5 Includes children between 6 and 23 months of age at baseline.

6 Significant effect at the 0.05 level.

7 Includes children between 24 and 35 months of age at baseline.

8 In the adjusted mixed-effects generalized linear regression models, treatment group, strata, baseline *z*-score, and child age were included as fixed effects and cluster as a random effect in all models, and wealth index was additionally included in the WAZ model.

Adjusted relative risks in the intention-to-treat population are presented in **Table 5**. Stunting prevalences decreased similarly over the 2 study years in both intervention and control children. The prevalence of underweight slightly declined and the prevalences of wasting and overweight remained low during follow-up, without any significant differences between groups, except for a lower prevalence of overweight and obesity in the intervention group at the year-2 follow-up. While the prevalence of breastfeeding naturally decreased over time as study children aged, we found a trend towards a higher proportion of breastfeeding in intervention children than in control children at the year-1 follow-up (*P* = 0.079). The proportion of children meeting minimum dietary diversity and the proportion consuming an iron-rich diet were both higher in intervention children than in control children at both the year-1 and year-2 follow-ups. At the year-1 follow-up, the proportion of children with reported diarrhea in the 2 weeks prior to the interview was lower in intervention children than in control children. For fever and LRTI, this was true at both the year-1 and year-2 follow-ups. More caregivers reported washing their hands with soap in the past 24 hours in the intervention group than in the control group at the year-1 follow-up. The per-protocol analyses showed similar results (**Supplemental Table 4**).

**TABLE 5:** Effects of the intervention bundle on child growth, feeding practices, and morbidity at 1- and 2-years after enrollment, using intention-to-treat analyses^1^

	Intervention *n* (%)	Control *n* (%)	Unadjusted RR (95% CI)	Adjusted RR (95% CI)
Stunting (height-for-age *z*-score less than −2.0)				
Baseline	218/984 (22%)	205/941 (22%)	1.02 (0.85–1.24)	1.03 (0.85–1.25)
Year-1 FU	172/901 (19%)	151/807 (19%)	1.03 (0.83–1.28)	1.01 (0.81–1.26)
Year-2 FU	112/862 (13%)	103/807 (13%)	1.02 (0.78–1.33)	1.00 (0.77–1.31)
Underweight (weight-for-age *z*-score less than −2.0)				
Baseline	96/984 (10%)	76/941 (8%)	1.21 (0.89–1.64)	1.23 (0.91–1.67)
Year-1 FU	75/902 (8%)	70/807 (9%)	0.97 (0.70–1.34)	0.96 (0.69–1.33)
Year-2 FU	41/863 (5%)	48/807 (6%)	0.78 (0.50–1.22)	0.78 (0.50–1.21)
Wasting (weight-for-height *z*-score less than −2.0)				
Baseline	17/984 (2%)	19/941 (2%)	0.85 (0.44–1.63)	0.89 (0.46–1.71)
Year-1 FU	21/902 (2%)	15/807 (2%)	1.28 (0.66–2.48)	1.30 (0.67–2.52)
Year-2 FU	15/860 (2%)	8/806 (1%)	1.76 (0.74–4.15)	1.79 (0.76–4.24)
Overweight or obese (weight-for-height *z*-score more than +2.0)				
Baseline	26/984 (3%)	19/941 (2%)	1.29 (0.71–2.34)	1.28 (0.71–2.32)
Year-1 FU	10/902 (1%)	11/807 (1%)	0.82 (0.35–1.94)	0.82 (0.35–1.94)
Year-2 FU	2/860 (0.2%)	9/806 (1.1%)	0.22 (0.05–1.05)	0.21 (0.05–0.99)^2^
Current breastfeeding^3,4^				
Baseline	361/561 (64%)	324/509 (64%)	1.00 (0.86–1.17)	1.01 (0.86–1.17)
Year-1 FU	67/154 (44%)	38/125 (30%)	1.42 (0.95–2.11)	1.43 (0.96–2.14)
Year-2 FU	NA^5^	NA	NA	NA
Meeting minimum dietary diversity^3,6^				
Baseline	211/561 (38%)	186/509 (37%)	1.04 (0.86–1.27)	1.04 (0.85–1.27)
Year-1 FU	75/154 (49%)	34/125 (27%)	1.81 (1.20–2.71)^2^	1.81 (1.20–2.72)^2^
Year-2 FU	681/863 (79%)	534/807 (66%)	1.19 (1.06–1.34)^2^	1.19 (1.06–1.33)^2^
Consuming an iron-rich diet^3,6^				
Baseline	111/559 (20%)	115/509 (23%)	0.89 (0.68–1.15)	0.89 (0.68–1.15)
Year-1 FU	102/154 (66%)	25/125 (20%)	3.33 (2.14–5.16)^2^	3.31 (2.13–5.13)^2^
Year-2 FU	595/863 (69%)	149/804 (19%)	3.71 (3.10–4.44)^2^	3.71 (3.10–4.44)^2^
Diarrhea in the past 2 weeks				
Baseline	239/980 (24%)	223/939 (24%)	1.03 (0.86–1.24)	1.02 (0.85–1.23)
Year-1 FU	117/901 (13%)	164/807 (20%)	0.64 (0.51–0.82)^2^	0.64 (0.50–0.81)^2^
Year-2 FU	36/863 (4%)	50/807 (6%)	0.66 (0.42–1.05)	0.66 (0.42–1.04)
Fever in the past 2 weeks				
Baseline	585/982 (60%)	559/940 (59%)	1.00 (0.89–1.13)	1.00 (0.89–1.13)
Year-1 FU	364/902 (40%)	455/806 (56%)	0.72 (0.62–0.82)^2^	0.71 (0.62–0.82)^2^
Year-2 FU	164/862 (19%)	241/807 (30%)	0.64 (0.52–0.78)^2^	0.64 (0.52–0.78)^2^
Lower respiratory tract infection in the past 2 weeks				
Baseline	150/936 (16%)	136/901 (15%)	1.05 (0.83–1.33)	1.05 (0.84–1.33)
Year-1 FU	150/901 (17%)	186/805 (23%)	0.72 (0.58–0.89)^2^	0.71 (0.57–0.88)^2^
Year-2 FU	38/862 (4%)	56/807 (7%)	0.65 (0.43–0.99)^2^	0.66 (0.44–0.99)^2^
Caregiver washing hands with soap in the past 24 hours				
Baseline	896/982 (91%)	822/939 (88%)	1.04 (0.95–1.15)	1.04 (0.95–1.14)
Year-1 FU	897/902 (99%)	708/804 (88%)	1.13 (1.02–1.25)^2^	1.13 (1.02–1.25)^2^
Year-2 FU	855/862 (99%)	177/806 (96%)	1.03 (0.93–1.13)	1.03 (0.93–1.13)

1 Unadjusted RRs were estimated using mixed-effects Poisson regression with treatment group and strata as fixed effects and cluster as a random effect. Of the prespecified baseline variables considered for inclusion in adjusted analyses (child age, child sex, whether the caregiver was the biological mother, caregiver's age, caregiver's education, caregiver's marital status, wealth index, household food insecurity index, number of household members, mean time to get water, drinking of safe water, and improved sanitation), only child age and child sex were included as additional fixed effects in multivariable analyses for adjusted RRs due to their significant predictions in bivariate analyses. The median child ages were 22.3 months (IQR, 14.8–29.5 months) at baseline, 34.3 months (IQR, 26.7–41.3 months) at year-1 FU, and 46.5 months (IQR, 38.9–53.6 months) at year-2 FU. Abbreviation: FU, follow-up.

2 Significant effect at the 0.05 level.

3 Assessed using the WHO and UNICEF guidelines on indicators for assessing infant and young child feeding practices.

4 Only children 6 to 23 months of age.

5 Not applicable.

6 Only children 6 to 23 months of age at baseline and year-1 FU, but all children at the year-2 FU (definition at year-2 FU for minimum dietary diversity was eating 4+ solid food groups).

**FIGURE 2:**
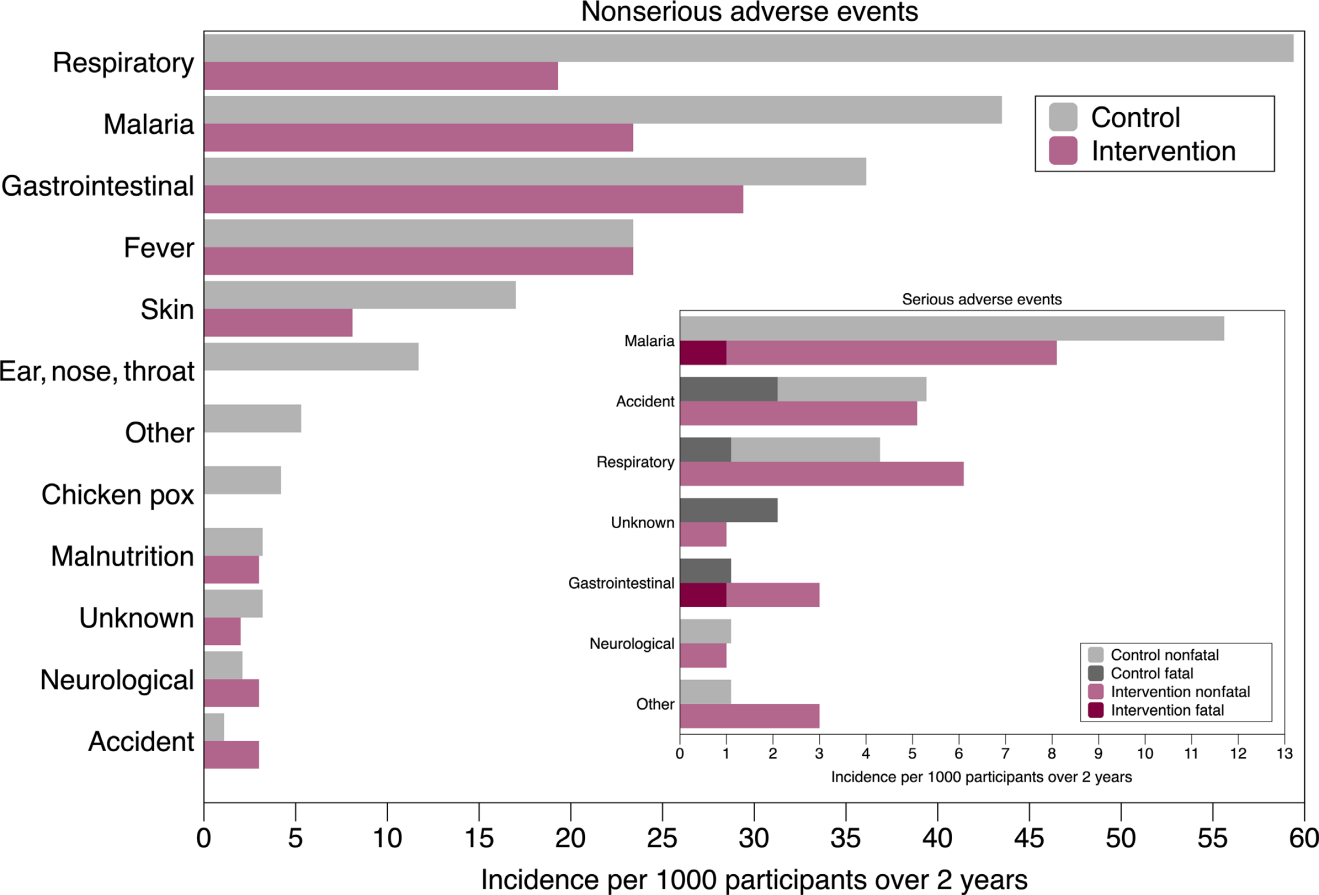
Adverse events by study group over the 2-year study period. Enrolled in control group, *n* = 942; enrolled in intervention group, *n* = 985. Adverse events were defined according to the International Conference on Harmonization (1996) E6 Guidelines for Good Clinical Practice. Any adverse event that resulted in death, was life threatening, required in-patient hospitalization, or resulted in persistent or significant disability or incapacity was defined as an SAE. All others were defined as nonserious adverse events. The adverse events were categorized as respiratory (pneumonia, cough, cold, flu, or breathing problems, with or without fever); gastrointestinal (diarrhea, with or without vomiting, with or without stomachache, with or without fever); fever (fever alone, or fever with headache and/or vomiting); skin (rashes, ringworms, boils, tungiasis); ear, nose, or throat (eye infection, ear infection, mouth candidiasis); neurological (convulsions, convulsive disorder); malnutrition [2 cases of moderate malnutrition (1 in each group) and 4 cases of severe acute malnutrition (WHZ less than −3 without edema; 2 in each group), all managed at the community level]; accident (burns, poisoning, road traffic accident, drowning, fall); other [cryptochidism, paraphimosis, septicemia or severe anemia (all SAEs), strabismus, swollen limbs, swollen scrotum, hemophilia]; or unknown (death with unknown cause; unclear symptoms, such as inability to walk, weakness). Abbreviations: SAE, serious adverse event; WHZ, weight-for-height *z*-score.

During the entire 2-year follow-up period, a total of 359 adverse events were reported: 221 in the control group and 138 in the intervention group. Of these, 52 (14.5%) were graded as serious (25 in the control group and 27 in the intervention group), and 8 resulted in death (6 in the control group and 2 in the intervention group). Only 1 nonserious adverse event was judged by the study clinician to be definitely related to the interventions: the participant was reported to have an animal protein allergy and reacted to egg administration, and was advised to stop intake after a follow-up hospital visit. Respiratory, malaria, and gastrointestinal infections were the leading causes of nonserious adverse events (**Figure 2**). Malaria was the leading cause of serious adverse events, followed by respiratory infection and accident (Figure 2). When serious and nonserious adverse events were combined, we found fewer children in the intervention than in the control group with respiratory (RR, 0.41; 95% CI: 0.25–0.69) and malaria (RR, 0.56; 95% CI: 0.33–0.95) infections (**Supplemental Table 5**). Three children (2 from the control group and 1 from the intervention group) were severely malnourished (WHZ less than −3 without bilateral edema) at the year-1 follow-up, and 1 child from the intervention group was severely malnourished at the year-2 follow-up. All were treated at the community level and recovered.

## Discussion

Our study showed a greater increase in HAZ (effect, 0.11; 95% CI: 0.02–0.19) over the entire 2-year follow-up period in the intervention group receiving agriculture, nutrition, and WASH interventions compared to the control group receiving agriculture interventions only. The intervention group also showed greater dietary diversity and a lower prevalence of morbidity during follow-up.

Compared to control children, we found larger effects on HAZ in intervention children over the entire 2-year follow-up and during the year-1 follow-up period, but not during the year-2 follow-up period. This, together with our subgroup analysis pointing towards the possibility that most of the benefit was for intervention children below 24 months of age at recruitment, suggests that age is an important effect modifier for such interventions. These findings are in line with the existing literature showing a progressive decrease in HAZ between ages 6–24 months in low- and middle-income countries (LMIC) (23); thus, ages below 24 months provide the best opportunity to intervene. The positive effect of combined nutrition and WASH interventions on HAZ when targeting 6- to 24-month-old children has been demonstrated in 3 recent randomized controlled trials—the WASH-Benefits trials in Bangladesh and Kenya and the SHINE (sanitation, hygiene, infant nutrition efficacy) trial in Zimbabwe (24–26)—which had effect sizes comparable to those of our study. These 3 trials all provided lipid-based nutrient supplements as the only nutrition-specific intervention, whereas we provided chickens to increase egg consumption, seeds to increase vegetable consumption, and MNP. Nonetheless, our study and the 3 referenced studies aimed to increase the intakes of calories, proteins, and micronutrients. Although the WASH interventions were more comprehensive in these other trials, as they included a sanitation component, the groups receiving only the WASH intervention did not show an effect on HAZ. This is in line with the results of most recent WASH publications, suggesting that more advanced WASH infrastructure, including provision of piped, safe water into households; human waste treatment facilities (27); and safe management of animal feces (28), are needed to see impacts on child growth. In contrast to the nutrition arm of the WASH-Benefits and SHINE trials, our study did not show an effect on the stunting prevalence. The stunting prevalence in our study decreased from 22% to 13% over the course of the study in both groups, which is likely related to the increasing age of enrolled children, as stunting naturally decreases with age (29,30), which has also been shown in Kenya (13).

Intervention children had a greater decrease in WHZ than control children during the entire 2-year follow-up, an effect not seen in the WASH-Benefits (25,26) and SHINE (24) trials. The effect might be transient and related to the smaller linear growth and the higher proportion of overweight children in the control group at the year-2 follow-up. Regardless, our results demonstrate no effect on wasting, comparable to the results of the aforementioned trials (24–26).

Improved dietary indicators may be 1 mechanism for the increase in HAZ seen in the intervention children in our study. At the end of the first year and during the entire 2-year follow-up period, the proportions of children meeting minimum dietary diversity and consuming iron-rich diets were higher in the intervention group compared to the control group. In our study, MNP provided an additional iron source, while the consumption of eggs and the provision of seeds to grow red onions and greens may have contributed to dietary variety. Further, the monthly nutrition and WASH group trainings may have triggered key behavior changes for improved child feeding practices. A recent study found that nutrition education and counselling through home visits and mother-peer groups improve infant and young child feeding practices (31). However, an effect of complementary feeding education on HAZ and stunting was only seen when such education was combined with the provision of complementary foods in food-insecure populations in LMIC (32). In our study, half of the households were severely food insecure and an additional 40% were moderately food insecure; therefore, the additional provision of eggs, MNP, and seeds in the intervention group might have contributed to improve linear growth.

Recent evidence on the causes of poor growth in young children points towards subclinical inflammation and environmental enteric dysfunction (23). To prevent infections, which cause such inflammation and enteric dysfunction, we provided chlorine, soap, and WASH training as part of our interventions. Morbidity caused by diarrhea, LRTI, and fever in the 2 weeks prior to reporting was lower in intervention than control children at follow-up. In contrast to our findings, the WASH-Benefits and SHINE trials found mixed effects of low-cost household WASH interventions on diarrhea (27). The clear reduction in the proportion of children with diarrhea in our study, from 24% at baseline to 13% at the year-1 follow-up in the intervention group, with no reduction in the control group, could be attributed to the WASH intervention, as has been demonstrated before (33). Potential mechanisms for the decrease in morbidity are that *1*) the enhanced diet including MNP increased the resistance to infections; *2*) the WASH interventions decreased the transmission of infectious pathogens; and *3*) appropriate treatment of diseases in our study population decreased the duration of infections in ill children. The proportions of children with diarrhea, fever, and LRTI sharply decreased at the end of the study in both groups, indicating that age may have an effect, but it could also be partly related to coronavirus disease 2019 (COVID-19) sensitization, as the endpoint assessment was conducted between mid-March and the end of April 2020, when COVID-19 reached Kenya, and strict hygienic measures were put in place that included soap distribution to all households, including those of control children. This is corroborated by a clear increase in caregivers washing their hands with soap in the past 24 hours in the control group between the year-1 and year-2 follow-ups, from 88% to 99%, but no change between baseline and the year-1 follow-up.

There are several possible reasons why the effects on linear growth were relatively small. The consumption of eggs, which we expected to have the biggest impact on growth, was lower than expected because the distributed chickens died rapidly and were not laying eggs as expected. Nonetheless, egg consumption was considerably higher in intervention than in control children but lower than in the studies providing eggs daily, showing effect sizes between 0.07 and 0.63 (8–10), as compared to 0.11 in our study. Despite training provided, the distribution of chickens to intervention households might have led to an increased risk of environmental enteric dysfunction due to increased contact with chicken feces, as indicated by a recent study in Western Kenya (34). Further, malaria is endemic in Western Kenya, with a prevalence of 15% in children in Kakamega county (35), and malaria was the second most reported adverse event in our study, which might have contributed to impaired linear growth in our study children. In comparison to other studies, many of our study participants were recruited at a late stage of or after transition from breastfeeding, which might have resulted in lower effects of the nutrition and WASH interventions. This is consistent with the lower changes in z-scores in the older children.

The intervention product costs were ${\$}$71 USD/year/child; when including delivery and staff costs, the annual cost per child was ${\$}$139 USD. This is in the range of cost estimates for programs aimed at managing child undernutrition in LMIC by non-health-care organizations (US${\$}$0.15–450 per child) (36), as well as product costs estimated in a research trial supplementing children with large- and medium-quantity lipid-based nutrient supplements (US${\$}$124 per child) to prevent malnutrition (37) while using small-quantity lipid-based nutrient supplements would result in lower costs (US${\$}$29 per child) (38). It is to be noted that costs could further decrease if the program were scaled up.

The strengths of this study include the provision of interventions that can be scaled relatively easily and the inclusion of the interventions into an existing platform that is likely to be sustainable. Moreover, we provided the interventions over 2 years, and reached high follow-up rates >85% in both groups. The study also had several limitations, including the limited number of eggs consumed by the participants, the lack of measurements for any biochemical indicators of nutritional status, the assessment of morbidity only quarterly by the 2-week cumulative prevalence, the lack of power to detect whether the subgroup differences by age and sex were true effects, and the study design not allowing for distinctions between the effects of the individual interventions on linear growth and morbidity. We did not adjust for multiple testing of secondary outcomes, and though these results need to be interpreted carefully, they enhance our understanding of the effects of the intervention (39).

In conclusion, providing a bundle of interventions, including agriculture, nutrition, and WASH products and behavior change trainings, resulted in a modest improvement in linear growth compared to the agriculture intervention alone. Our observation that younger children that are 6–23 months of age at recruitment may benefit more from the interventions suggests value in more targeted provision of interventions to children younger than 2 years. In addition, the high prevalences of poverty and food insecurity globally (32) and specifically in the study area underscore the need for a multisectoral approach including WASH, but also education, with strong behavior change approaches needed to tackle malnutrition.

## Supplementary Material

nqac098_Supplemental_File

## Data Availability

Deidentified data described in the manuscript, code book, and analytic code will be made available upon request pending application and approval.

## Notes

The Children's Investment Fund Foundation, United Kingdom, provided funds to One Acre Fund for the intervention implementation (grant # 1606-01373) and to GroundWork for the evaluation part of the study (grant # 1703-01854).

The funder of the study contributed to discussions during the study design and conducted field visits during the data collection, but was not involved during the data analysis, data interpretation, or manuscript writing.

Supplemental Methods and Supplemental Tables 1–5 are available from the “Supplementary data” link in the online posting of the article and from the same link in the online table of contents at https://academic.oup.com/ajcn/.

BAW and FR contributed equally to this work as senior authors.

Abbreviations used: COVID-19, coronavirus disease 2019; HAZ, height-for-age *z*-score; LMIC, low- and middle-income countries; LRTI, lower respiratory tract infection; MNP, micronutrient powders; ORS, oral rehydration solution; SHINE, sanitation, hygiene, infant nutrition efficacy project; WASH, water, sanitation, and hygiene; WAZ, weight-for-age *z*-score; WHZ, weight-for-height *z*-score.
