# Supplementary material for: Effectiveness of an integrated agriculture, nutrition-specific, and nutrition-sensitive program on child growth in Western Kenya: a cluster-randomized controlled trial
Source: Am J Clin Nutr. 2022 Apr 14;116(2):446–59. doi: 10.1093/ajcn/nqac098 (PMC9348977; doi:10.1093/ajcn/nqac098)
Supplement: nqac098_Supplemental_File [file nqac098_supplemental_file.zip › OSM supplementary Table 1_220310.pdf]

Supplementary Table 1: Average change in z-scores for intervention and control children during both years of follow-up, during the year 1 follow-up, and during the year 2 follow-up, using per-protocol analyses [1]

	n [2]	Unadjusted mean change (95% CI)	n	Unadjusted mean change (95% CI)	Effect (95% CI)	Adjusted mean change (95% CI)		Effect (95% CI)
	Intervention		Control			Intervention	Control	
**Change HAZ**								
Both years	568	0.29 (0.22, 0.37)	807	0.17 (0.10, 0.24)	**0.12 (0.02, 0.23)** [3]	0.31 (0.24, 0.38)	0.16 (0.10, 0.22)	**0.15 (0.06, 0.24)**
Year 1	595	0.11 (0.05, 0.16)	807	0.06 (0.01, 0.11)	0.05 (-0.03, 0.12)	0.12 (0.07, 0.17)	0.05 (0.01, 0.09)	0.07 (-0.001, 0.13)
Year 2	540	0.13 (0.07, 0.20)	745	0.10 (0.05, 0.16)	0.03 (-0.06, 0.11)	0.14 (0.07, 0.20)	0.10 (0.05, 0.16)	0.03 (-0.05, 0.11)
**Change in WAZ**								
Both years	568	0.02 (-0.03, 0.08)	807	-0.03 (-0.08, 0.02)	0.06 (-0.02, 0.13)	0.02 (-0.03, 0.07)	-0.03 (-0.07, 0.01)	0.05 (-0.01, 0.12)
Year 1	595	-0.07 (-0.11, -0.02)	807	-0.10 (-0.14, -0.06)	0.04 (-0.02, 0.10)	-0.07 (-0.11, -0.02)	-0.10 (-0.14, -0.07)	0.04 (-0.02, 0.09)
Year 2	540	0.05 (0.01, 0.09)	745	0.07 (0.04, 0.11)	-0.02 (-0.08, 0.03)	0.05 (0.01, 0.09)	0.08 (0.04, 0.11)	-0.03 (-0.08, 0.03)
**Change in WHZ**								
Both years	566	-0.13 (-0.19, -0.07)	806	-0.09 (-0.14, -0.04)	-0.04 (-0.11, 0.04)	-0.14 (-0.19, -0.09)	-0.09 (-0.13, -0.04)	-0.05 (-0.12, 0.02)
Year 1	595	-0.14 (-0.20, -0.09)	807	-0.16 (-0.21, -0.11)	0.01 (-0.06, 0.09)	-0.15 (-0.20, -0.10)	-0.15 (-0.20, -0.11)	0.004 (-0.06, 0.07)
Year 2	538	-0.003 (-0.05, 0.04)	744	0.07 (0.03, 0.11)	**-0.07 (-0.14, -0.01)**	-0.01 (-0.05, 0.04)	0.07 (0.03, 0.11)	**-0.08 (-0.14, -0.02)**

HAZ, height-for-age z-score; WAZ, weight-for-age z-score; WHZ, weight-for-height z-score.

[1] For the per-protocol analyses the children in the lowest tercile of the adherence index based on PCA analysis for each time period were excluded. Mixed effects generalized linear models with treatment group and strata as fixed effects and cluster as random effect were used to compare the unadjusted average change in the intervention and the control groups during the follow-up period. In the adjusted analyses, we included baseline z-score as a fixed factor in all models. In addition, of the pre-specified baseline variables considered for inclusion in adjusted analyses (child age, child sex, whether caregiver was biological mother, caregiver's age, caregiver's education, caregiver's marital status, wealth index, household food insecurity index, number of household members, mean time to get water, drinking of safe water, improved sanitation), only child age, and child sex were included as fixed effects in multivariable analyses for HAZ and WHZ due to their significant prediction of change in HAZ and WHZ in bivariate analyses. For WAZ, wealth index was additionally included as a fixed effect as it was a significant predictor in bivariate analyses.

[2] Slight difference in numbers between different z-scores are due to some flagged values that were excluded for analysis.

[3] Bold font indicates a significant effect at the 0.05 level.
