# Supplementary material for: Effectiveness of an integrated agriculture, nutrition-specific, and nutrition-sensitive program on child growth in Western Kenya: a cluster-randomized controlled trial
Source: Am J Clin Nutr. 2022 Apr 14;116(2):446–59. doi: 10.1093/ajcn/nqac098 (PMC9348977; doi:10.1093/ajcn/nqac098)
Supplement: nqac098_Supplemental_File [file nqac098_supplemental_file.zip › OSM supplementary Table 2_220314.pdf]

Supplementary Table 2: Sensitivity analyses for height-for-age z-score for intervention and control children during follow-up, using different approaches to intention-to-treat analyses [1]

	n	Unadjusted mean (95% CI)	n	Unadjusted mean (95% CI)	Effect (95% CI)	Adjusted mean (95% CI)		Effect (95% CI)
	**Intervention**		**Control**			**Intervention**	**Control**	
**Exclusion of all outliers in change in HAZ of ≤-4 and ≥4**								
Both years	853	0.20 (0.15, 0.26)	805	0.17 (0.12, 0.23)	0.03 (-0.05, 0.11)	0.22 (0.17, 0.26)	0.16 (0.11, 0.20)	0.06 (-0.01, 0.13)
Year 1	898	0.10 (0.05, 0.15)	804	0.05 (0.002, 0.10)	0.05 (-0.02, 0.12)	0.11 (0.06, 0.15)	0.05 (0.001, 0.09)	0.06 (-0.00003, 0.12)
Year 2	809	0.10 (0.06, 0.15)	745	0.11 (0.06, 0.15)	-0.001 (-0.06, 0.06)	0.11 (0.07, 0.15)	0.10 (0.06, 0.15)	0.003 (-0.06, 0.06)
**Recoding of all otuliers in change of HAZ: <- 4 recoded to -4 and >4 recoded to 4**								
Both years	862	0.24 (0.18, 0.30)	807	0.17 (0.11, 0.25)	0.07 (-0.02, 0.15)	0.25 (0.20, 0.31)	0.16 (0.10, 0.21)	**0.10 (0.02, 0.17)** [2]
Year 1	901	0.11 (0.07, 0.16)	807	0.06 (0.01, 0.11)	0.06 (-0.01, 0.13)	0.12 (0.07, 0.16)	0.05 (0.004, 0.10)	**0.07 (0.002, 0.13)**
Year 2	816	0.12 (0.07, 0.17)	745	0.11 (0.06, 0.16)	0.01 (-0.06, 0.08)	0.12 (0.08, 0.17)	0.10 (0.06, 0.15)	0.02 (-0.05, 0.08)
**Comparison of mean HAZ at endpoint**								
Year 2 FU	862	-0.79 (-0.88, -0.71)	807	-0.94 (-1.03, -0.85)	**0.15 (0.02, 0.27)**	-0.82 (-0.87, -0.76)	-0.92 (-0.98, -0.86)	**0.11 (0.02, 0.19)**

FU, follow-up; HAZ, height-for-age z-score

[1] Mixed effects generalized linear models with treatment group and strata as fixed effects and cluster as random effect were used to compare the unadjusted average change and HAZ at endpoint in the intervention and the control groups during the follow-up period. In the adjusted analyses, we included baseline HAZ as a fixed factor in all models. In addition, of the pre-specified baseline variables considered for inclusion in adjusted analyses (child age, child sex, whether caregiver was biological mother, caregiver's age, caregiver's education, caregiver's marital status, wealth index, household food insecurity index, number of household members, mean time to get water, drinking of safe water, improved sanitation), only child age, and child sex were included as fixed effects in multivariable analyses due to their significant prediction of change in HAZ and HAZ at endpoint.

[2] Bold font indicates a significant effect.
