# Supplementary material for: Effectiveness of an integrated agriculture, nutrition-specific, and nutrition-sensitive program on child growth in Western Kenya: a cluster-randomized controlled trial
Source: Am J Clin Nutr. 2022 Apr 14;116(2):446–59. doi: 10.1093/ajcn/nqac098 (PMC9348977; doi:10.1093/ajcn/nqac098)
Supplement: nqac098_Supplemental_File [file nqac098_supplemental_file.zip › OSM supplementary Table 3_220309.pdf]

Supplementary Table 3: Unadjusted mean z-scores for intervention and control children at baseline and at 1- and 2-years post enrolment [1]

	n [2]	Mean (SD)	n	Mean (SD)
	**Intervention**		**Control**	
**Height-for-age z-score**				
Baseline	984	-1.06 (1.36)	941	-1.13 (1.28)
Year 1 FU	901	-0.95 (1.28)	807	-1.05 (1.18)
Year 2 FU	862	-0.79 (1.18)	807	-0.94 (1.08)
**Weight-for-age Z score**				
Baseline	984	-0.56 (1.14)	941	-0.59 (1.07)
Year 1 FU	902	-0.63 (1.01)	807	-0.69 (0.97)
Year 2 FU	863	-0.56 (0.93)	807	-0.61 (0.90)
**Weight-for-height Z score**				
Baseline	984	-0.02 (0.99)	941	-0.02 (0.97)
Year 1 FU	902	-0.18 (0.90)	807	-0.17 (0.91)
Year 2 FU	860	-0.16 (0.84)	806	-0.10 (0.88)

FU, follow-up; median child age at baseline was 22.3 (IQR 14.8-29.5) months, at year 1 FU 34.3 (26.7-41.3) and at year 2 FU 46.5 (38.9-53.6).

[1] Data are mean (SD).

[2] Slight difference in numbers between different z-scores are due to some flagged values that were excluded from analysis.
