# Supplementary material for: Effectiveness of an integrated agriculture, nutrition-specific, and nutrition-sensitive program on child growth in Western Kenya: a cluster-randomized controlled trial
Source: Am J Clin Nutr. 2022 Apr 14;116(2):446–59. doi: 10.1093/ajcn/nqac098 (PMC9348977; doi:10.1093/ajcn/nqac098)
Supplement: nqac098_Supplemental_File [file nqac098_supplemental_file.zip › OSM supplementary Table 4_220310.pdf]

Supplementary Table 4: Effect of the intervention bundle on child growth, feeding practices, and morbidity at 1- and 2-years post enrolment, using per-protocol analyses [1]

	Intervention n (%)	Control n (%)	Unadjusted RR (95% CI)	Adjusted RR (95% CI)
**Stunting (height-for-age Z score less than -2.0)**				
Baseline	137/656 (21%)	205/941 (22%)	0.97 (0.78, 1.20)	0.98 (0.79, 1.21)
Year 1 FU	108/595 (18%)	151/807 (19%)	0.98 (0.77, 1.26)	0.99 (0.77, 1.26)
Year 2 FU	76/568 (13%)	103/807 (13%)	1.05 (0.78, 1.41)	1.04 (0.77, 1.40)
**Underweight (Weight-for-age Z score less than -2.0)**				
Baseline	62/656 (9%)	76/941 (8%)	1.17 (0.83, 1.66)	1.19 (0.84, 1.69)
Year 1 FU	43/595 (7%)	70/807 (9%)	0.86 (0.59, 1.26)	0.86 (0.59, 1.26)
Year 2 FU	23/568 (4%)	48/807 (6%)	0.67 (0.39, 1.13)	0.66 (0.39, 1.13)
**Wasting (Weight-for-height Z score less than -2.0)**				
Baseline	8/656 (1%)	19/941 (2%)	0.60 (0.26, 1.36)	0.61 (0.27, 1.40)
Year 1 FU	12/595 (2%)	15/807 (2%)	1.16 (0.54, 2.48)	1.17 (0.55, 2.51)
Year 2 FU	11/566 (2%)	8/806 (1%)	2.00 (0.80, 5.00)	2.05 (0.82, 5.11)
**Overweight or obese (Weight-for-height Z score more than +2.0)**				
Baseline	16/656 (2%)	19/941 (2%)	1.21 (0.62, 2.35)	1.22 (0.63, 2.38)
Year 1 FU	4/595 (0.7%)	11/807 (1.4%)	0.49 (0.16, 1.55)	0.49 (0.16, 1.56)
Year 2 FU	2/566 (0.4%)	9/806 (1.1%)	0.34 (0.07, 1.58)	0.32 (0.07, 1.51)
**Current breastfeeding [2]**				
Baseline	220/366 (60%)	324/509 (64%)	0.93 (0.79, 1.11)	0.95 (0.80, 1.13)
Year 1 FU	38/90 (42%)	38/125 (30%)	1.34 (0.85, 2.11)	1.34 (0.86, 2.11)
Year 2 FU	NA [3]	NA	NA	NA
**Meeting minimum dietary diversity [4]**				
Baseline	133/366 (36%)	186/509 (37%)	1.02 (0.81, 1.27)	1.01 (0.81, 1.26)
Year 1 FU	43/90 (48%)	34/125 (27%)	**1.75 (1.11, 2.76)** [5]	**1.75 (1.11, 2.76)**
Year 2 FU	453/568 (80%)	534/807 (66%)	**1.20 (1.06, 1.37)**	**1.20 (1.06, 1.36)**
**Consuming an iron-rich diet [4]**				
Baseline	78/365 (21%)	115/509 (23%)	0.96 (0.72, 1.28)	0.96 (0.72, 1.27)
Year 1 FU	61/90 (68%)	25/125 (20%)	**3.36 (2.10, 5.36)**	**3.33 (2.08, 5.33)**
Year 2 FU	392/568 (69%)	149/804 (19%)	**3.72 (3.08, 4.50)**	**3.72 (3.08, 4.50)**
**Diarrhoea in past 2 weeks**				
Baseline	149/653 (23%)	223/939 (24%)	0.96 (0.78, 1.18)	0.95 (0.78, 1.17)
Year 1 FU	69/594 (12%)	164/807 (20%)	**0.58 (0.43, 0.77)**	**0.58 (0.44, 0.77)**
Year 2 FU	19/568 (3%)	50/807 (6%)	**0.54 (0.31, 0.93)**	**0.54 (0.31, 0.93)**
**Fever in past 2 weeks**				
Baseline	387/654 (59%)	559/940 (59%)	0.99 (0.87, 1.13)	1.99 (0.87, 1.13)
Year 1 FU	245/595 (41%)	455/806 (56%)	**0.73 (0.62, 0.85)**	**0.73 (0.62, 0.85)**
Year 2 FU	112/568 (20%)	241/807 (30%)	**0.66 (0.53, 0.82)**	**0.66 (0.52, 0.82)**
**Cough in past 2 weeks**				
Baseline	100/620 (16%)	136/901 (15%)	1.04 (0.80, 1.35)	1.04 (0.80, 1.35)
Year 1 FU	106/594 (18%)	186/805 (23%)	**0.76 (0.60, 0.96)**	**0.76 (0.60, 0.97)**
Year 2 FU	21/568 (4%)	56/807 (7%)	**0.54 (0.33, 0.89)**	**0.54 (0.33, 0.89)**
**Caregiver washing hands with soap in past 24 hours**				
Baseline	600/654 (92%)	822/939 (88%)	1.05 (0.94, 1.17)	1.05 (0.94, 1.17)
Year 1 FU	591/595 (99%)	708/804 (88%)	**1.13 (1.01, 1.26)**	**1.13 (1.01, 1.26)**
Year 2 FU	564/567 (99%)	777/806 (96%)	1.03 (0.92, 1.15)	1.03 (0.92, 1.15)

FU, follow-up.

[1] For the per-protocol analysis, the children in the lowest tercile of the adherence index based on principal component analysis were excluded. Unadjusted RR were estimated using mixed effects Poisson regression with treatment group and strata as fixed effects and cluster as random effect. Of the pre-specified baseline variables considered for inclusion in adjusted analyses (child age, child sex, whether caregiver was biological mother, caregiver's age, caregiver's education, caregiver's marital status, wealth index, household food insecurity index, number of household members, mean time to get water, drinking of safe water, improved sanitation), only child age and child sex were included as additional fixed effects in multivariable analyses for adjusted RR due to their significant prediction in bivariate analyses.

[2] Only children 6-23 months of age.

[3] Not applicable, as children had grown out of the breastfeeding age range.

[4] Only children 6-23 months of age at baseline and 1-year FU, but all children in at 2-year FU (definition at year 2 FU for minimum dietary diversity: ate 4+ solid food groups).

[5] Bold font indicates a significant effect at the 0.05 level.
