# Supplementary material for: Effectiveness of an integrated agriculture, nutrition-specific, and nutrition-sensitive program on child growth in Western Kenya: a cluster-randomized controlled trial
Source: Am J Clin Nutr. 2022 Apr 14;116(2):446–59. doi: 10.1093/ajcn/nqac098 (PMC9348977; doi:10.1093/ajcn/nqac098)
Supplement: nqac098_Supplemental_File [file nqac098_supplemental_file.zip › OSM supplementary Table 5_220310.pdf]

Supplementary Table 5: Incidence of combined serious and non-serious adverse events by study group and relative risk [1]

	Intervention		Control		RR (95% CI)
**Adverse event category**	**n**	**Incidence per 1000 participants over 2 years**	**n**	**Incidence per 1000 participants over 2 years**	
Respiratory [2]	25/985	25.4	60/942	63.7	**0.41 (0.25, 0.68)** [3]
Malaria	31/985	31.5	52/942	55.2	**0.56 (0.33, 0.95)**
Gastrointestinal [4]	32/985	32.5	35/942	37.2	0.88 (0.52, 1.47)
Fever [5]	23/985	23.4	22/942	23.4	0.96 (0.44, 2.10)
Skin [6]	8/985	8.1	16/942	17.0	0.49 (0.20, 1.21)
Ear, nose, throat [7]	0/985	0	11/942	11.7	..
Accident [8]	8/985	8.1	6/942	6.4	1.27 (0.44, 3.70)
Chicken pox	0/985	0	4/942	4.2	..
Malnutrition [9]	3/985	3.0	3/942	3.2	0.96 (0.19, 4.74)
Neurological [10]	4/985	4.1	3/942	3.2	0.90 (0.11, 7.11)
Other [11]	3/985	3.0	6/942	6.4	0.32 (0.06, 1.71)
Unknown	3/985	3.0	5/942	5.3	0.59 (0.13, 2.73)

[1] Mixed effects Poisson regression was used to compare incidences of adverse events, with treatment group as fixed effect and cluster as random effect.
[2] Includes pneumonia, cough, cold, flu, or breathing problems, with or without fever.
[3] Bold font indicates a significant effect at the 0.05 level.
[4] Includes diarrhea with or without vomiting, with or without stomachache, with or without fever.
[5] Includes fever alone, or fever with headache and/or vomiting.
[6] Includes rashes, ringworms, boils, tungiasis.
[7] Includes eye infection, ear infection, mouth candidiasis.
[8] Includes burns, poisoning, road traffic accident, drowning, fall.
[9] Includes 2 cases of moderate malnutrition (1 in each group) and 4 cases of severe acute malnutrition (WHZ<-3 without edema; 2 in each group).
[10] Includes convulsion, convulsive disorder.
[11] Includes cryptochidism, paraphimosis, septicemia or severe anemia, strabismus, swollen limbs, swollen scrotum, hemophilia.
