# Supplementary material for: Effectiveness of an integrated agriculture, nutrition-specific, and nutrition-sensitive program on child growth in Western Kenya: a cluster-randomized controlled trial
Source: Am J Clin Nutr. 2022 Apr 14;116(2):446–59. doi: 10.1093/ajcn/nqac098 (PMC9348977; doi:10.1093/ajcn/nqac098)
Supplement: nqac098_Supplemental_File [file nqac098_supplemental_file.zip › OSM_methods_220323.pdf]

Supplementary methods

Section **Page**

Random allocation of misfits …………………………………………………….. 1

Nutrtion and WASH group trainings ……………………………………………... 1

Method description and training schedule …………………………………… 1

Training modules on intervention product use and growth monitoring ………... 3

Intervention product demonstrations ……………………………………. 4

Growth monitoring and promotion ……………………………………… 11

Behavior change communictiation on growth monitoring and promotion ... 17

Takeaway card for MNP, chlorine, ORS and zinc use ………………………… 23

Measurement of adherence to study interventions…………………………………… 24

References ………………………………………………………………………… 24

Random allocation of misfits

We used the user-written *randtreat* command in Stata to assign clusters to the intervention or control group, and specified the option *misfits(global)*, which takes all misfits and randomly allocates them to either the intervention or control group, so that balance between the intervention and control group is prioritized rather than balance between strata (1). In practice, there were 9 strata, and 4 of them had an odd number of clusters, resulting in one misfit each after randomly allocating the rest to either intervention or control group. These 4 misfits were then randomly assigned so that 2 were intervention and 2 were control. This maintained the overall balance of 63 clusters per arm, but also only resulted in an imbalance of only 1 cluster each in these 4 strata.

Nutrition and WASH group trainings

Method description and training schedule

The training and behavior change communication component in the intervention group was delivered by One Acre Fund Health Field Officers. Every month, Health Field Officers met with groups of approximately twenty caregivers from one or multiple clusters depending on their size. They met in a convenient place within their cluster or a neighboring one.

The training package covered a range of nutrition-specific and nutrition-sensitive topics (**Figure 1**). Each topic was covered in two consecutive sessions: the first one focused on technical knowledge, and the second one was dedicated to dialogue and counselling. Both, technical lessons and dialogue counselling, followed the same structure, including attendance check, energizer, refreshers, activity, and commitments. Additionally, during the first session,

trainers provided key technical messages (technical lesson) followed by an activity to put the theory in practice. The following month, a second session was dedicated to a discussion (dialogue counselling) around two stories: a problem story and a positive action story. The stories were taught by the trainers, using A3 illustrations organized in a flipbook. The problem story aimed to trigger conversations about the importance of a specific health/nutrition issue in the participants' community. After the positive action story, participants were probed to point out the differences between the two stories and identify their own barriers and enablers to the promoted behaviors. The trainer's role was to guide the participants to identify root causes of the issues in question and share ideas or experience on concrete solutions. At the end of the discussion, participants shared a commitment related to the discussed behaviors.

Given the long period over which the training and behavior change intervention was delivered, including refreshers for previous topics in every session was essential in ensuring participants retained key messages. Additionally, every six months, a session was dedicated to a topic that the participants and the trainers wanted to review.

In terms of materials, trainers were provided with printed training guides, storybooks, job aids, tools for the activities and a tablet, with which they recorded participants' attendance and takeaways from the session.

The training package was developed in collaboration with the Kenya Ministry of Health and aligned with their recommendations. The demand from the caregivers and the priority public health issues in the targeted communities were also considered. Once developed, the training content and illustrations were adjusted to the local context based on field tests.

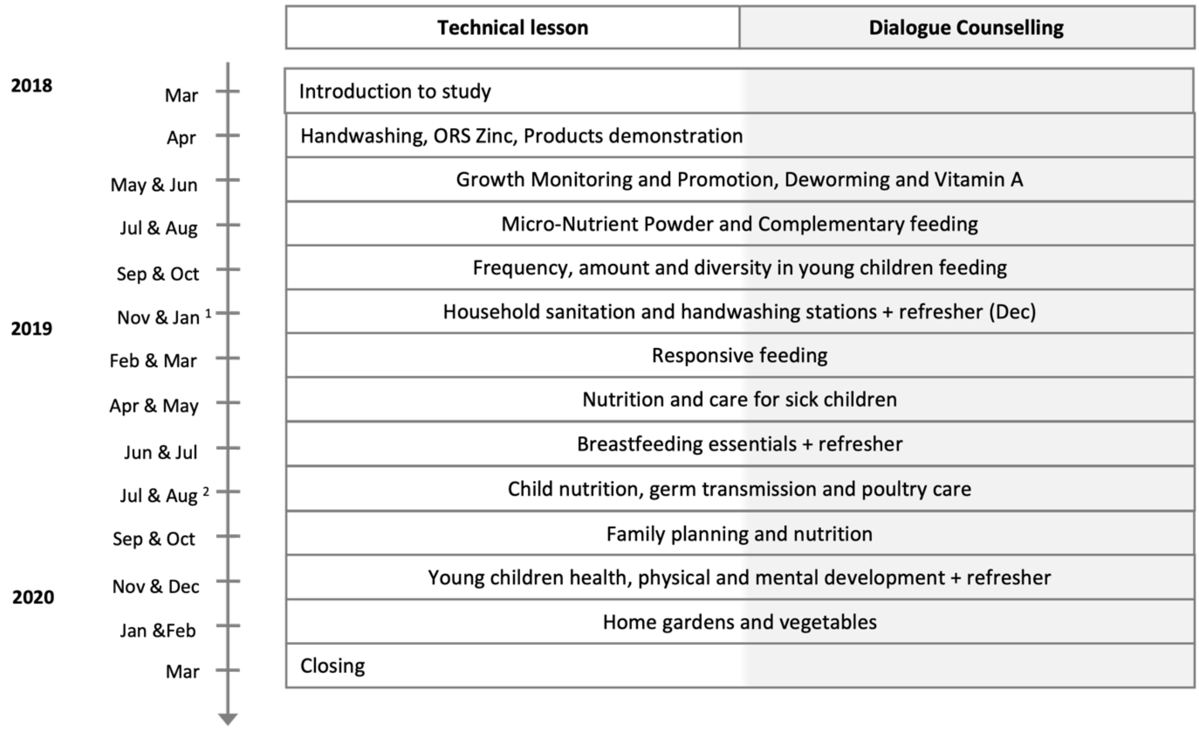

Figure 1: Monthly nutrition and WASH training schedule

[1] In December 2018 only a short refresher training was conducted due to the Christmas break. The technical lesson on the respective topic was taught in November and the dialogue counselling was conducted in January.

[2] Since the sessions were taking place every four weeks (during intervention product distribution), we had one session starting at the beginning of the month of July and one at the end of the month of July 2019.

Training modules on intervention product use and growth monitoring

As an example the training modules 2 to 4 are included in the following pages.

Total time: 110 minutes

1. Welcome & Objectives (5 Minutes)

SAY: Today we will:

1. Talk about the importance of handwashing to prevent diarrhea and illness.
2. Learn how to correctly wash hands.
3. Learn how to give ORS and zinc to children with diarrhea.
4. Practice adding MNP to food.

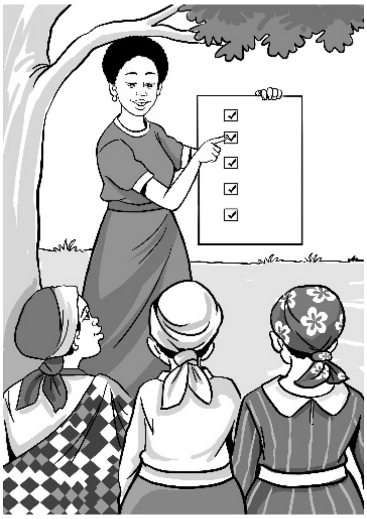

SAY: Our key messages for this lesson are:

1. Everyone should wash their hands with soap and water at 5 critical times:
    1) After using the latrine
    2) After changing a nappy or handling feces
    3) Before preparing food
    4) Before eating or feeding a child
    5) After handling a sick person
2. Handwashing with soap and water is one of the most effective ways to prevent diarrhea and child malnutrition.
3. Give Oral Rehydration Salts (ORS) and zinc tablets to children with diarrhea to help them get better faster.
4. Continue giving breastmilk, food, and clean water to children who have diarrhea.

Materials

DO: Make sure you have everything you will need for this lesson:

- Attendance sheet with section for feedback notes on barriers
- 1 sachet of MNP
- ½ liter of previously treated water in container with lid
- Soap and clean water for handwashing
- 1 portion of uji in a container with lid

2. Attendance (5 Minutes)

DO:

1. Complete Attendance Sheet and ask about those who are missing.
2. Request that those who came go and visit those who did not to tell them about the lesson. Ask for volunteers and commitments to do so.
3. Thank the participants for their hard work and encourage them to continue.
    - Encourage them to have a sense of pride that they are protecting themselves, their children against illness and malnutrition.

3. Game – Two Birds[1] (10 Minutes)

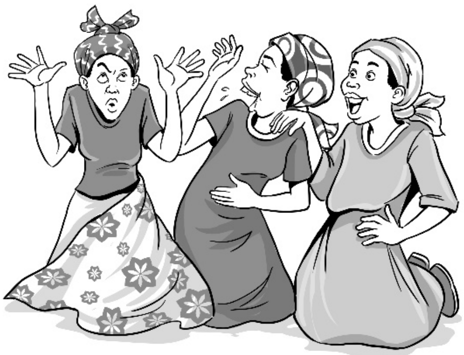

1. Ask participants to form a circle all facing inward.
2. Explain that they are to imagine two kinds of birds.
3. If you say 'soar' then the participants should imitate a large eagle, arms out wide like wings and stand on the tips of their toes to indicate that they are very high up in the sky.
4. Explain that when they hear 'cluck', they should imitate a chicken, squatting or bending down and flapping their elbows at their sides.
5. Take turns calling, 'soar', and 'cluck', getting faster with each round to see who can keep up without doing the incorrect movement.

SAY: Now that we are energized, let's begin our lesson.

4. Review and Troubleshoot (30 Minutes)

SAY: Let's begin by reviewing last month's lesson!

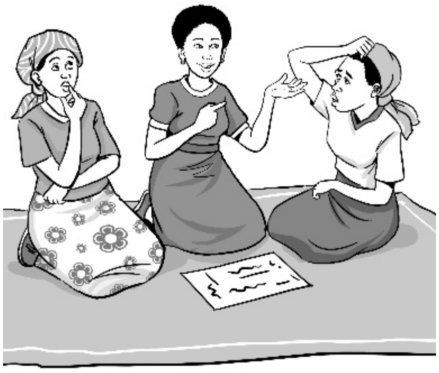

? Who can name the topics we taught last month?

- *importance of nutrients and MNP for young children*
- *how to add MNP to a child's food*
- *how to care for poultry*
- *importance of young children eating eggs*
- *how to treat drinking water with liquid chlorine*

? How often should your young child eat MNP?

- *every 3 days*

? What are the benefits of a child eating MNP?

- *helps protect against sickness*
- *helps child grow tall and strong*
- *helps brain development so child performs better in school*

? Why is it important to keep chickens contained in an area away from children?

- *chicken poop contains diseases that can make young children very sick or even die, so chickens need to be kept separate from anywhere children can go*

? How much chlorine is needed to treat one jerry can of water?

- *1 capful*

? How much chlorine is needed to treat one liter of water?

- *3 drops*

[1] 101 Ways to energizer groups: Games to use in workshops, meetings and the community. Available from www.aidsalliance.org International HIV/AIDS Alliance. 2002.

? How long do we need to wait before drinking water treated with chlorine?

- *30 minutes, but if there is a chlorine taste then wait 2 hours and the taste will be gone.*

SAY: Practicing new behaviors can be difficult. Let's talk about what made it difficult to practice the behaviors from last month.
[Trainers, after the meeting write a summary of these difficulties to report to 1AF]

? Who found it difficult to **give their child MNP every three days**?

- What made it difficult for you?
    - What would we make it easier for you?
        - How can we make that happen for you?
            - Can we agree that you will try…?
- Did anyone else find this difficult? (*repeat the questions until everyone's barriers have been address and solutions proposed*)

? Who found it difficult to **treat all your drinking water with chlorine**?

- What made it difficult for you?
    - What would we make it easier for you?
        - How can we make that happen for you?
            - Can we agree that you will try…?
- Did anyone else find this difficult?

? Who found it difficult to **give your child one egg every day laid by your chickens**?

- What made it difficult for you?
    - What would we make it easier for you?
        - How can we make that happen for you?
            - Can we agree that you will try…?
- Did anyone else find this difficult?

? Who found it difficult to **keep chickens contained in an area separate from places children go**?

- What made it difficult for you?
    - What would we make it easier for you?
        - How can we make that happen for you?
            - Can we agree that you will try…?
- Did anyone else find this difficult?

? Do you have any other questions or challenges from last month's lesson?

5. Technical Lesson (30 Minutes)

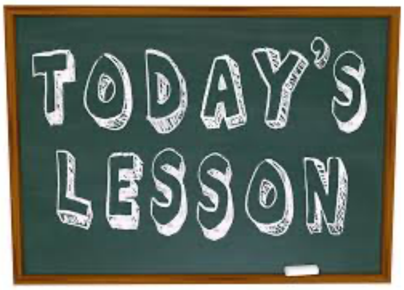

SAY:

? What have you heard about handwashing?

- How should we wash our hands?
- When should we wash our hands?
- Why should we wash our hands?

? If children do get diarrhea, how should we care for them?

1. **Handwashing with soap and water** is one of the most effective ways to prevent diarrhea and child malnutrition.
   - Everyone should wash their hands at the **five critical times**
     1. After using the latrine
     2. After changing a nappy or handling feces
     3. Before preparing food
     4. Before eating or feeding a child
     5. After touching a sick person

2. **Handwashing with soap kills more germs than water alone.**
   - If there is no soap, ash can also be used.
   - Hands should be scrubbed for at least 20 seconds then rinsed.
   - Put handwashing stations with soap near the latrine and eating areas to help family members remember to wash hands.

3. **Oral Rehydration Salts (ORS)** should be given to children with diarrhea to help the child get better faster.
   - **ORS** is a powder that should be mixed into clean water and given to children with diarrhea to help them recover.

SAY: Now we will learn the 4 STEPS to use Oral Rehydration Salts (ORS)

1. Boil or treat ½ liter of water and pour into a clean container.
2. Mix one sachet of ORS with the ½ liter of clean water.
3. Encourage child to drink as much as possible from a clean cup. Continue making more as diarrhea lasts.
4. Continue providing ORS and zinc for 10-14 days. If the child does not improve, take him to a health center.

- Continue giving as much ORS as the child will drink until the diarrhea stops.
- If diarrhea does not stop after 3-4 days, take the child to a health facility or health worker.
- Drinking ORS will not increase diarrhea or make it worse; it will help the diarrhea stop sooner and keep the child from loosing too much water.
- Unused ORS drink should be discarded after 24 hours.

4. **Zinc tablets** should be given with ORS to children with diarrhea to help the child get better faster.
   - Give 1 zinc tablet per day as diarrhea starts for 10 to 14 days.
   - Continue providing ORS and zinc for 10-14 days. If diarrhea does not stop after 3-4 days, take the child to a health facility or health worker.

5. **Continue giving breastmilk and (if older than 6 months) food and clean water** to children with diarrhea. Take 1 zinc tablet per day while diarrhea continues.
   - Breastfeed more often during illness to help the child recover faster and not lose as much weight.
   - Take time to patiently encourage your sick child to eat as her/his appetite may be decreased because of the illness.
   - Encourage the child to eat small frequent meals throughout the day. This is important to maintain the child’s strength and reduce weight loss.
   - Sick children may need extra encouragement to eat.

? Does anyone have any questions?

6. Activity (20 Minutes)

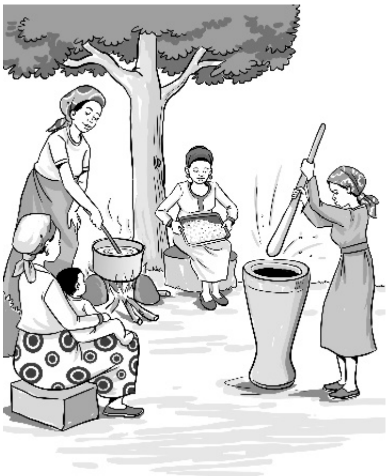

SAY: Let's practice what we just learned! We will demonstrate adding ORS to water as we would for children with diarrhea.

DO: Bring ½ liter of previously treated water.

SAY:

1. Boil or treat with chlorine water and let cool.
2. Using clean spoon, mix one sachet of ORS with the ½ liter of clean water.
3. Encourage the sick child to drink as much as possible from a clean cup and make more as needed.

SAY: Now we'll demonstrate how to add MNP to food like we learned last month.

ASK: Who can remember the 5 steps of using MNP?

DO: Review with the group, trying to get them to offer the information rather than you telling them.

HOW TO USE MNP: *5 STEPS*

1. Wash your hands and your child's hands with soap and water.
2. Prepare food for your child (like uji), and **let it cool enough to eat.**
   - Do **NOT** add the MNP to liquid (tea) or very hot foods.
   - Pour food into a clear bowl with a clean spoon.
3. Use a clean spoon to push a portion of the food to the side of the bowl.
4. Take a small sachet (1g), tear it open, and **add it to the portion of the prepared food.**
5. Mix the food well, and serve it to your child immediately.
   - First feed the child the portion that contains MNP.
   - Make sure your child consumes the food **within 30 minutes** of opening the sachet.
   - Only use **1 sachet per child**, **do not share** – you have enough for 1 child for a month!

DO: Demonstrate how to add MNP to the thick uji you brought.

- Remind participants that the bowl and spoon are clean.
- Show the thick consistency of the uji. Food for children over 6 months is thick enough if it does not drip off the spoon.
- Show how you separate a portion of the food in order to ensure the child eats the MNP. Add the MNP to this portion.

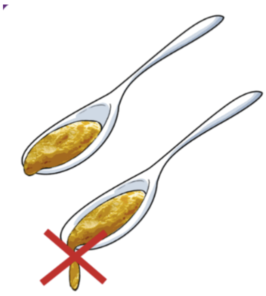

ASK: Does anyone have questions about handwashing, diarrhea, ORS, zinc, or MNP?

7. Closing and Review (10 Minutes)

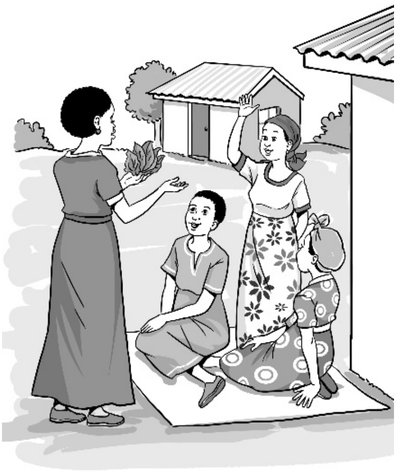

SAY: Let's finish by reviewing what we learned today.

? When are the 5 critical times to wash hands with soap and water?

1. *After using the latrine*
2. *After changing a nappy or handling feces*
3. *Before preparing food*
4. *Before eating or feeding a child*
5. *After touching a sick person*

? What happens if mothers and children do not wash their hands at these times?

- *Children may become malnourished, sick with diarrhea, or even die.*

? What should you do when your child has diarrhea?

- *Give ORS mixed into clean water until diarrhea stops.*
- *Give 1 zinc tablet per day as diarrhea starts for 10 to 14 days.*
- *Continue breastfeeding.*
- *If over 6 months, give extra food and water. Sick children may need extra encouragement to eat.*

DO: Thank everyone for coming and remind them about the date and time of next month's meeting.

DO: Distribute products to the caregivers in the meeting.

Total time: 90 minutes

1. Welcome & Objectives (5 Minutes)

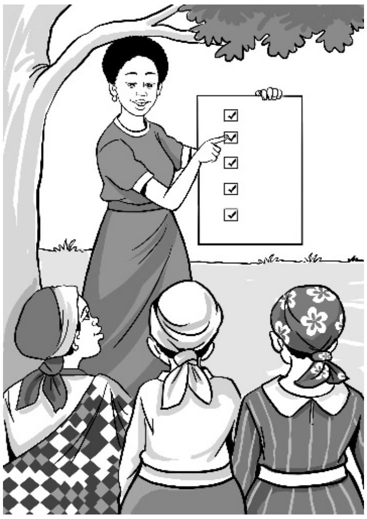

SAY: Today we will:

1. Discuss the importance of Growth Monitoring and Promotion (GMP) services.
2. Learn why children need to go to health facilities for vaccinations and deworming.
3. Find out the importance of vitamin A supplementation for children during Malezi Bora (Child Health Week).

SAY: Our key messages for this lesson are:

1. **Monthly growth monitoring** will help identify child health problems early and prevent malnutrition.
2. Vitamin A supplementation improves immunity and helps prevent diseases, diarrhea and malnutrition.
3. Children should receive **deworming medicine** and **vitamin A supplements** every 6 months to help prevent malnutrition.

Materials

DO: Make sure you have everything you will need for this lesson:

- Attendance sheet with section for feedback notes on barriers
- 1 flipchart
- 2+ markers in 2+ colors
- Counseling card #38
- Monitoring booklet with growth monitoring page

2. Attendance (5 Minutes)

DO:

1. Complete Attendance Sheet and ask about those who are missing.
2. Request that those who came go and visit those who did not to tell them about the lesson. Ask for volunteers and commitments to do so.
3. Thank the participants for their hard work and encourage them to continue.
   - Encourage them to have a sense of pride that they are protecting themselves, their children against illness and malnutrition.

3. Game – Sinking Islands [1] (10 Minutes)

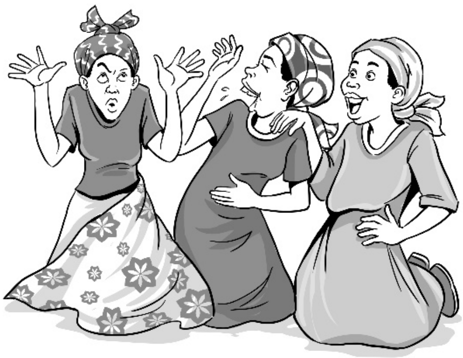

1. Draw five shapes on the floor with chalk, tape or in the sand large enough for all of the participants to stand in.
2. Have the participants name the islands and describe a few qualities of the islands. For example, one may have waterfalls, one has mountains, one has desert, one has modern buildings and one has a lot of animals.
3. Ask the participants to choose the island where they would like to live and have them go to that island.
4. Explain that the islands will sink one at a time and when their island sinks, they must move as quickly as possible to the next island. The leader calls out which island is sinking and advises the participants to evacuate.
5. Play continues until all of the islands have sunk and all the participants are on one single island.

SAY: Now that we are energized, let's begin our lesson.

4. Review and Troubleshoot (30 Minutes)

SAY: Let's begin by reviewing last month's lesson!

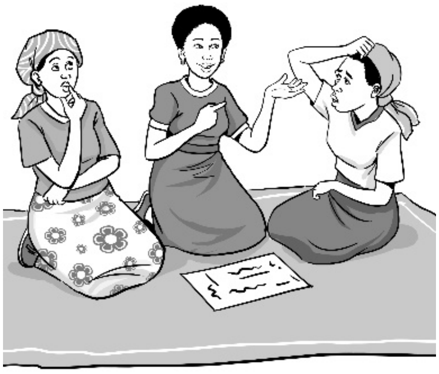

? Who remembers the topics we covered last month?

- *Wash hands with soap at 5 critical times.*
- *Give ORS and zinc tablets to children with diarrhea to help them get better faster.*
- *Continue giving breastmilk, food, and clean water to children who have diarrhea.*
- *How to prepare ORS for children with diarrhea.*
- *How to prepare MNP for the children in the study.*

? When are the 5 critical times to wash hands with soap and water?

1. *After using the latrine*
2. *After changing a nappy or handling feces*
3. *Before preparing food*
4. *Before eating or feeding a child*
5. *After touching a sick person*

? What happens if mothers and children do not wash their hands at these times?

- *Children may become malnourished, sick with diarrhea, or even die.*

[1] 101 Ways to energizer groups: Games to use in workshops, meetings and the community. Available from www.aidsalliance.org International HIV/AIDS Alliance. 2002.

? What should you do when your child has diarrhea?

- *Give ORS mixed into clean water until diarrhea stops.*
- *Give 1 zinc tablet per day as diarrhea starts for 10 to 14 days.*
- *Continue breastfeeding.*
- *If over 6 months, give extra food and water. Sick children may need extra encouragement to eat.*

SAY: Practicing new behaviors can be difficult. Let's talk about made it difficult to practice the behaviors from last month.
[Trainers, after the meeting write a summary of these difficulties to report to 1AF]

? Who found it difficult to wash **their hands and their children's hands with soap at the 5 critical times**?

- What made it difficult for you?
    - What would we make it easier for you?
        - How can we make that happen for you?
            - Can we agree that you will try…?
- Did anyone else find this difficult? [*repeat the questions until everyone's barriers have been address and solutions proposed*]

? Who found it difficult to **give children with diarrhea ORS, zinc tablets, and extra food**?

- What made it difficult for you?
    - What would we make it easier for you?
        - How can we make that happen for you?
            - Can we agree that you will try…?
- Did anyone else find this difficult?

? Who found it difficult to **give your child MNP every other day**?

- What made it difficult for you?
    - What would we make it easier for you?
        - How can we make that happen for you?
            - Can we agree that you will try…?
- Did anyone else find this difficult?

? Do you have any other questions or challenges from last month's lesson?

5. Technical Lesson (15 Minutes)

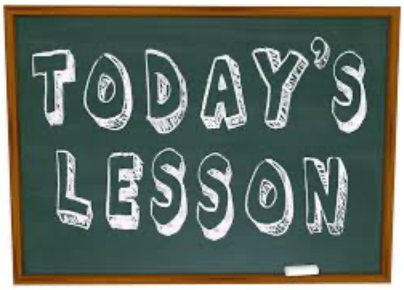

SAY:

? What have you heard about growth monitoring and promotion (GMP) at the health facility?

? What have you heard about vitamin A?

? What have you heard about deworming?

Identifying malnutrition early saves children's lives. Growth monitoring will help keep your child healthy.

1. **Monthly growth monitoring** will help identify child health problems early and prevent malnutrition.
    - At growth monitoring, your child should be measured to help you know if your child is tall enough for his age.
    - At growth monitoring, your child will be weighed to help you know if your child is heavy enough for his height.
    - All children under 5 years of age should go to health facilities for monthly growth monitoring.

2. **Vitamin A** supplementation will help children grow well and avoid being sick.
    - It helps prevent diarrhea which leads to malnutrition.
    - Vitamin A also helps the immunizations your child receives work better to protect him from all kinds of illnesses.
    - Children should get vitamin A every six months starting at six months old.
    - Mothers should take children to health facilities during Malezi Bora (Child Health Week) twice each year.

3. **Once children begin to eat food** and put things in their mouths, they are more likely to get infected with worms.
    - Worms take all the nutritional value from food a child eats and make it more likely that a child will become malnourished.
    - Children should get deworming treatment very six months beginning at one year old.

4. **Children will be given** vitamin A and deworming medications when they go to growth monitoring services at the health facility.

? Does anyone have any questions?

6. Activity (15 Minutes)

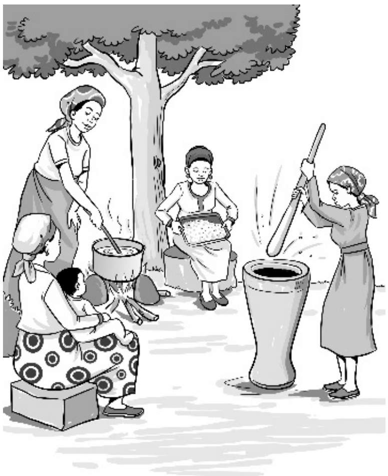

SAY: Let's practice what we just learned!

- The purpose of the activity is to help us remember the key messages from the lesson and important things in the story.
- We are going to participate in an activity to understand how growth monitoring services work.

DO:

- Present counseling cards #38 and discuss the images of the child being weighed and measured.
- Show the participants the growth monitoring card and explain where they should want to see their child's measure range on the chart.
- Draw it out on a flip chart and explain that the green lines mean the child is in the proper growth range.
- Explain that the red lines and pink shading mean that the child is not in the proper range.
- Explain that the women should see a new mark in their child's health book every month.

SAY:

- How many of you have taken their child for growth monitoring?
- Can one or two volunteers explain their experiences?
- Have you brought your child's health card?

DO:

- Examine the child health cards together.
- Praise the women for attending growth monitoring, even if their child is not in the healthy range.

SAY:

- Who has a child in the healthy range?
- What advice would you give to women whose children are not in the healthy range?

DO: If deworming and vitamin A are not mentioned,

- Remind participants why these are important in fighting malnutrition.
- Show them where the vitamin A and deworming should be marked in their cards.
- Summarize by reminding participants that malnutrition can be cured, but it is easiest and causes less damage when it is identified very early.

SAY:

We have all learned so much! You are doing a very good job of learning how to keep children in your communities healthy.

? Are there any questions?

7. Review and Closing (10 Minutes)

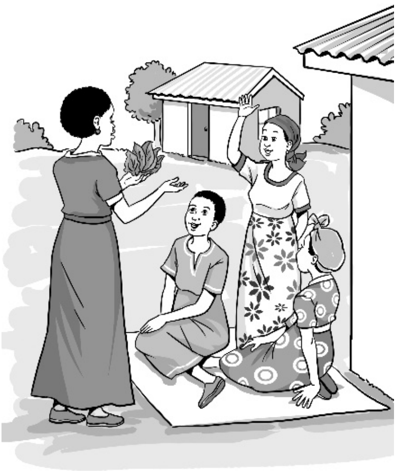

SAY: Let's finish by reviewing what we learned today.

? What is the purpose of growth monitoring?

- *Children's weight and height are measured to see if they are tall enough and heavy enough for their age.*

? What medicines will your child receive during Malezi Bora?

- *Vitamin A supplementation and deworming.*

? How often should your child receive vitamin A and deworming tablets?

- *Twice per year*

? Why should children take vitamin A supplements twice per year?

- *It helps prevent diseases, diarrhea and malnutrition.*

? Why should children take deworming tablets twice per year?

- *Worms in the body steal the nutrients children eat, which makes children malnourished.*

DO: Thank everyone for coming and remind them about next month's meeting.

DO: Distribute products to the caregivers in the meeting.

Total time: 95 minutes

1. Welcome & Objectives (5 Minutes)

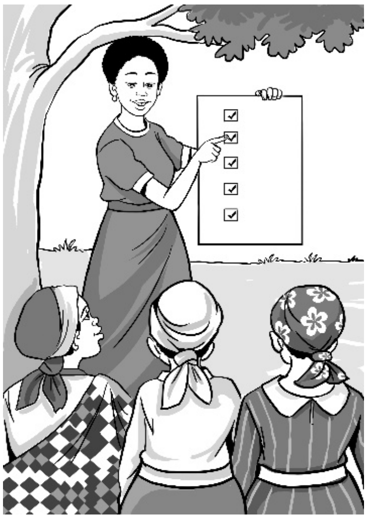

SAY: Today we will:

1. Find out what makes it difficult for caregivers to take their children to growth monitoring services (GMS).
2. Find out what makes it difficult for caregivers to take their child to receive vitamin A.
3. Find out what makes it difficult for caregivers to take their child to receive deworming.
4. Talk about solutions to those problems.
5. Do an activity to help us remember the lesson.
6. Make commitments to try new actions.

SAY: Our key messages for this lesson are:

1. **Monthly growth monitoring** will help identify child health problems early and prevent malnutrition.
2. Vitamin A supplementation improves immunity and helps prevent diseases, diarrhea and malnutrition.
3. Children should receive **deworming medicine** and **vitamin A supplements** every 6 months to help prevent malnutrition.

Materials

DO: Make sure you have everything you will need for this lesson:

- Attendance sheet with section for feedback notes on barriers
- Storybook set for Lesson 4 (Stories 1 and 2)
- Scenarios sheets for GMP role play

2. Attendance (5 Minutes)

DO:

1. Complete Attendance Sheet and ask about those who are missing.
2. Request that those who came go and visit those who did not to tell them about the lesson. Ask for volunteers and commitments to do so.
3. Thank the participants for their hard work and encourage them to continue.
    - Encourage them to have a sense of pride that they are protecting themselves, their children against illness and malnutrition.

3. Game – Relay Counting[1] (10 Minutes)

[1] 101 Ways to energizer groups: Games to use in workshops, meetings and the community. Available from www.aidsalliance.org International HIV/AIDS Alliance. 2002.

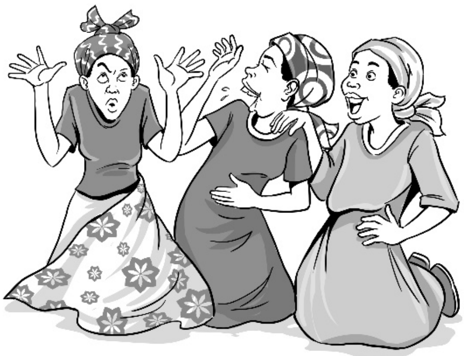

1. Participants sit or stand in a circle.
2. The group will count from 1 to 7. Each time a person calls out a number, they raise a hand. If they raise their right hand, then the person on their right continues in sequence, but if they raise their left hand, the person on their left continues in sequence.
3. Once the number 7 is announced, the next person, either on the right or the left should start over with 1.
4. Play continues until the group is lively from laughing at their mistakes.

SAY: Now that we are energized, let's begin our lesson.

4. Review and Troubleshoot (10 Minutes)

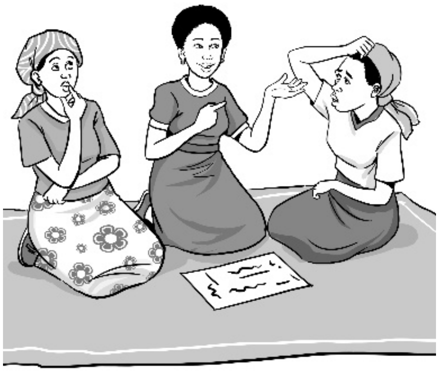

SAY: Let's begin by reviewing last month's lesson!

? Who remembers the topics we covered last month?

- *Growth monitoring.*
- *Vitamin A supplementation and deworming during Malezi Bora (child health week).*

? What is the purpose of growth monitoring?

- *Children's weight and height are measured to see if they are tall enough and heavy enough for their age.*

? What medicines will your child receive during Malezi Bora?

- *Vitamin A supplementation and deworming.*

? How often should your child receive vitamin A supplements and deworming tablets?

- *Twice per year.*

? Why should children take vitamin A supplements twice per year?

- *It helps prevent diseases, diarrhea and malnutrition.*

? Why should children take deworming tablets twice per year?

- *Worms in the body steal the nutrients children eat, which makes children malnourished.*

? Do you have any questions or challenges from last month's lesson?

5. Storytelling (45 Minutes)

SAY: To help us remember last month's lesson on growth monitoring and to understand the information better, we are going to read and discuss some stories together.

1. Problem Story

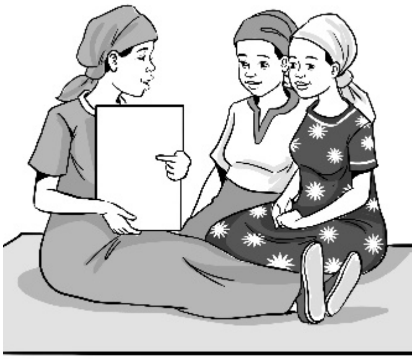

DO: Read the **problem story** from the flipbook. Remember to use the storytelling skills that were presented in the training.

Read the questions in the flipbook and lead the participants in a discussion about this story. Wait for responses after reading the question. Try not to suggest answers; let the participants give the answers and express their opinions.

2. Positive Action Story

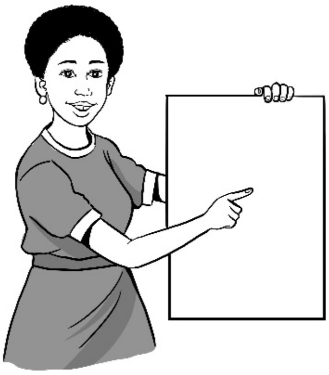

SAY: We've already seen a story about some choices that resulted in sad or unhappy consequences. Let's look now at some different choices and actions.

DO: Read the **positive action story** set using the same techniques as the first. Ensure that the story is well understood.

3. Identify Barriers and Enablers

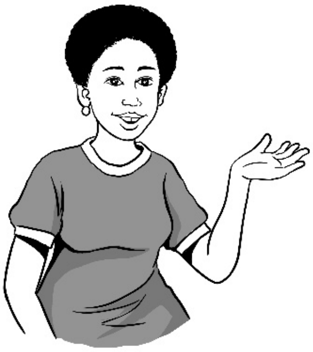

DO: Ask the questions at the end of the positive story. Discuss how this story might be similar or different from the last story. Identify barriers and enablers by talking through these questions.

The trainer's goal is to identify the 'root causes' of problems and get down to the 'lowest doable actions'. Then, explore solutions and negotiate behaviors.

Make a mental note or write down what the participants say and summarize these after the training.

6. Activity (20 Minutes)

SAY: Let's practice what we just learned!

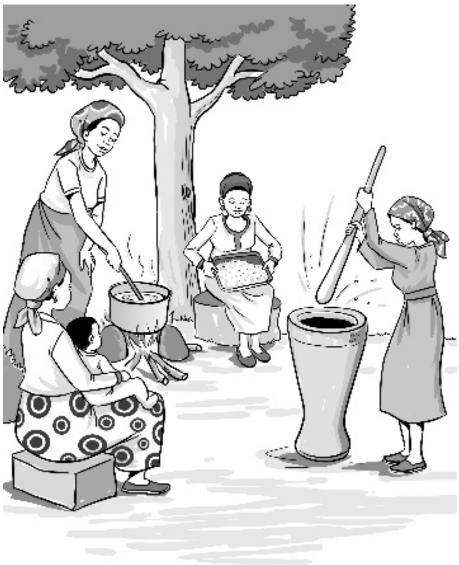

- The purpose of the activity is to help us remember the key messages from the lesson and important things in the story.
- We are going to participate in an activity to understand why growth monitoring services is crucial for your child's development.

Groups of 4
Explain the scenario to each group.
1 piece of paper: chart + icons + child age

SAY:
- We are going to do a role play by groups.
- Divide into groups of four people.
- Each participant of the group picks a role:
  - 2 health workers
  - 2 care givers of under 5 children

DO:
- Distribute to each group a scenario.

SAY:
- Let's pretend that the caregivers visit the health workers with their child because of the situation described on the sheet. There is a completed growth monitoring chart and indications corresponding to a scenario (child age, nutrition status…).
- I will pass in each group to explain the scenario.
- The goal is for the health workers to understand the situation, identify the good practices or problems to advice the care giver accordingly.
- You will have 10 minutes to discuss.
  - Firstly, the health workers should describe and explain the growth monitoring chart to the care givers.
    - *Is the child in the health range? Be careful, the lower line is for girls and the upper line is for boys.*
    - *What is the direction of the line?*
    - *What does it imply for the child health?*
  - Secondly, the health workers have to question the care givers to identify problems or good practices that led to the situation described on the growth monitoring chart. Here are some examples, ask all the questions you want!
    - *How old is the child?*
    - *Have you exclusively breastfed your child until 6 months of age?*
    - *Are you still breastfeeding?*

        - *What kind of food does the child usually eat?*
        - *What make it difficult to give enough quantity and variety of food to your child?*
    - Finally, the health worker will counsel the care givers on how to overcome the barriers or praise good practices. Don't hesitate to discuss all together in the group to identify the key messages health workers should give to caregivers. Think about the positive action story we just discussed.

DO:

- Visit each group and explain the scenario, answer questions from participants if needed.
- Circulate among the groups while they discuss.
- After 10 minutes, ask participants to get ready for debriefing.

SAY:

- Let's talk all together about your scenarios and what the health workers advised to the care givers.
    - Group 1, can you explain your situation? How did you counsel the care givers?

DO:

- Ask the same questions to each group for debriefing. Make the group agree on key counseling messages for each scenario.
- If deworming and vitamin A are not mentioned,
    - Remind participants why these are important in fighting malnutrition.
    - Show them where the vitamin A and deworming should be marked in their cards.
- Summarize by reminding participants that malnutrition can be cured, but it is easiest and causes less damage when it is identified very early.

SAY:

We have all learned so much! You are doing a very good job of learning how to keep children in your communities healthy.

? Are there any questions?

7. Commitment Circle and Closing (5 Minutes)

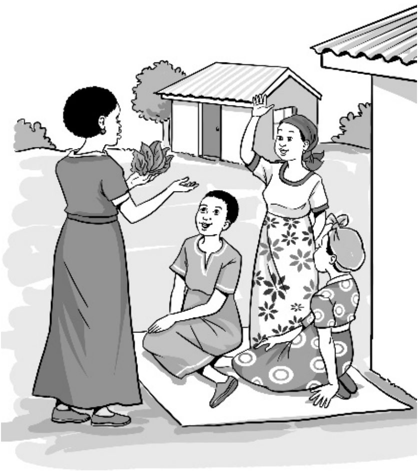

SAY: Today we will finish the meeting with a special commitment ceremony. Please come and join me in a circle because we are about to close our meeting. Thank you very much for your participation and your hard work today.

SAY: We are now going to take turns going around the circle to share 1 or more commitments that we are willing to make based on what we have learned today. Let us please take a moment to decide what our commitments will be, and to share them with the group.

DO: Remember that when a participant shares a commitment, it may not be the complete action that has been promoted in the lesson, but encourage her/him, support her/him and thank her/him for her contribution.

- Clap and celebrate!

DO: Thank everyone for coming and remind them about next month's meeting.

DO: Distribute products to the caregivers in the meeting.

Takeaway card for MNP, chlorine, ORS and zinc use

FEED CHILDREN MICRONUTRIENT POWDER (MNP)

ONE ACRE FUND

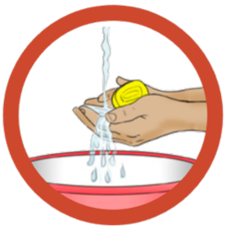

Wash your hands and your child's hands with soap and water before preparing food!

1 Prepare food for child in clean bowl. Let it cool.

2 Use a clean spoon to push a **portion** of the food to the side of the bowl.

3 Add **1 MNP sachet** to that portion only.

4 Mix well and feed the child **immediately** with the **MNP portion first**.

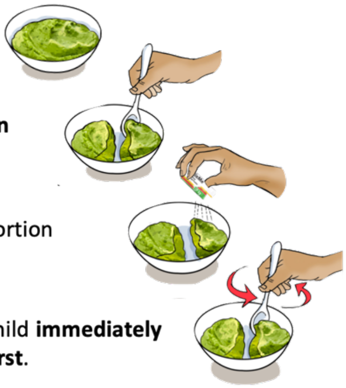

5 Child must eat the food **within 30 minutes** of opening the sachet!

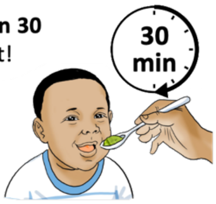

DO NOT ADD TO LIQUID OR HOT FOODS
DO NOT SHARE

1 sachet every 3 days for 1 child							
Day	1	2	3	4	5	6	7

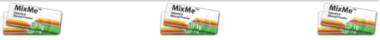

PURIFY WATER

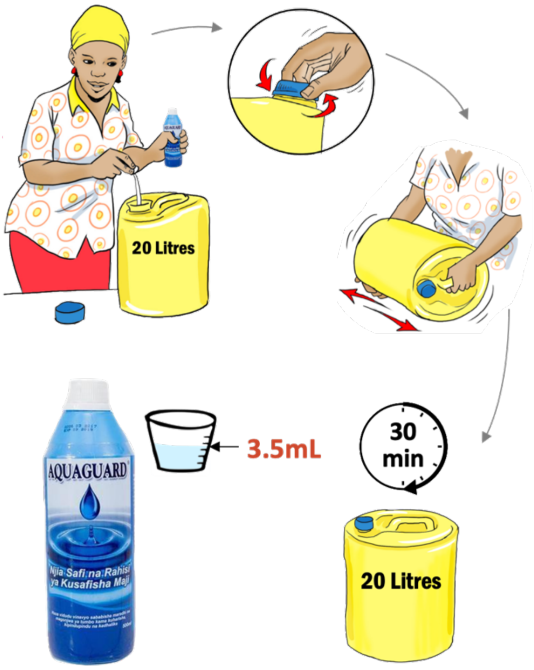

Instructions are also available on the bottle.

TREAT DIARRHEA

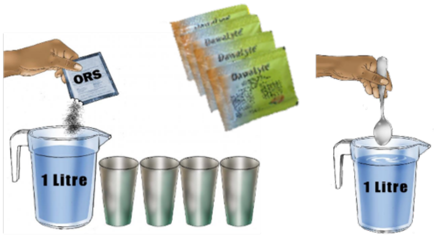

- Encourage child to drink as much as possible from a clean cup.
- Continue providing ORS until diarrhea stops. **If the child does not improve after 3-4 days, take her/him to a health center.**

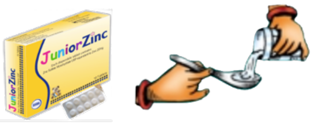

- Dissolve 1 tablet of zinc in 1 teaspoon of breastmilk or clean water.
- Give 1 zinc tablet every day for **10-14 days**.

Measurement of adherence to study interventions

We measured adherence as receipt of six physical items distributed to the households of intervention children during quarterly visits. Chlorine bottles were weighed at each visit to determine the amount of chlorine solution used in the prior quarter. Every 6 months, each household received 550 g of chlorine solution, so the intended chlorine use was 275 g per quarter. The mean number of grams of chlorine solution used per quarter during the follow-up period was calculated as the adherence indicator for this product. Caretakers were instructed to save the empty MNP packets which were counted at each quarterly visit. The adherence indicator was the total number of MNP sachets consumed by the study child during the follow-up period. Study personnel replaced used MNP sachets to ensure that 10 sachets were available to each intervention child at each monthly visit. As a result, the maximum number of MNP sachets which could be consumed by each child was 10 per month, or 240 during the two years of follow-up. Interviewers asked caretaker respondents about child egg consumption, and the adherence indicator was the mean number of eggs eaten per week during the follow-up period. Caretakers were instructed to feed intervention children one egg per day. Inspection of households' soap proved to be difficult, so the adherence indicator for use of soap distributed by study personnel was the number of quarters soap was reported as received. Because the ultimate objective of distributing onion and greens seeds was consumption of the resulting crop by household members, the adherence indicator was the number of years these crops were consumed in the study household.

Although ORS and zinc supplementation tablets were distributed to intervention children, they were meant to be used only during diarrheal disease episodes. The proportion of diarrhoeal episodes for which the child received ORS and/or zinc was calculated, but not included in the combined adherence index because of the relatively small number of children in the denominator. Moreover, because the number of diarrhoea cases is considered a dependent variable and as such, this number changes over time as a result of the intervention. In addition, in order to measure exposure to these interventions among children in the control group, identical or similar questions were asked during interviews conducted of caregivers with the same frequency as that in the intervention group.

The number of nutrition and WASH trainings attended by the caregiver or a representative was not included in the adherence index as this is considered an intermediate measure that should result in the improvement in other adherence indicators and secondary study outcomes.

All six of these adherence indicators were calculated separately for follow-up year one and follow-up year two, as well as both follow-up years together. The indicators were combined using principal component analysis into an adherence index calculated for each intervention child's household. Separate adherence index calculations were done for intervention children for whom the primary outcome of change in HAZ could be calculated between enrolment and the end of two years of follow-up, for intervention children for whom change in HAZ could be calculated between enrolment and the end of the first year follow-up, and for children for whom change in HAZ could be calculated between the end of the first year of follow-up and the end of the second year follow-up. Per-protocol analyses were done after excluding intervention children who were in the lowest tercile of the appropriate adherence index.

References

1. Carril A. Dealing with misfits in random treatment assignment. Stata J. 2017;17:652–67.
